# TAB1 regulates glycolysis and activation of macrophages in diabetic nephropathy

**DOI:** 10.1007/s00011-020-01411-4

**Published:** 2020-10-12

**Authors:** Hanxu Zeng, Xiangming Qi, Xingxin Xu, Yonggui Wu

**Affiliations:** Department of Nephropathy, The First Affiliated Hospital of Anhui Medical University, No. 218, Jixi Rd., Hefei, Anhui China

**Keywords:** Diabetic nephropathy, Glucose metabolism, Macrophage, TAB1

## Abstract

**Objective and design:**

Macrophages exhibit strong phenotypic plasticity and can mediate renal inflammation by polarizing into an M1 phenotype. They play a pivotal role in diabetic nephropathy (DN). Here, we have investigated the regulatory role of transforming growth factor β-activated kinase 1-binding protein 1 (TAB1) in glycolysis and activation of macrophages during DN.

**Methods:**

TAB1 was inhibited using siRNA in high glucose (HG)-stimulated bone marrow-derived macrophages (BMMs) and lentiviral vector-mediated TAB1 knockdown was used in streptozotocin (STZ)-induced diabetic mice. Western blotting, flow cytometry, qRT-PCR, ELISA, PAS staining and immunohistochemical staining were used for assessment of TAB1/nuclear factor-κB (NF-κB)/hypoxia-inducible factor-1α (HIF-1α), iNOS, glycolysis, inflammation and the clinical and pathological manifestations of diabetic nephropathy.

**Results:**

We found that TAB1/NF-κB/HIF-1α, iNOS and glycolysis were up-regulated in BMMs under HG conditions, leading to release of further inflammatory factors, Downregulation of TAB1 could inhibit glycolysis/polarization of macrophages and inflammation in vivo and in vitro. Furthermore, albuminuria, the tubulointerstitial damage index and glomerular mesangial expansion index of STZ-induced diabetic nephropathy mice were decreased by TAB1 knockdown.

**Conclusions:**

Our results suggest that the TAB1/NF-κB/HIF-1α signaling pathway regulates glycolysis and activation of macrophages in DN.

**Electronic supplementary material:**

The online version of this article (10.1007/s00011-020-01411-4) contains supplementary material, which is available to authorized users.

## Introduction

It has been widely accepted that inflammation is central to the development of diabetic nephropathy (DN). This disorder of glucose control in diabetics can cause inflammation and cellular stress, causing functional deterioration seen in DN [1, 2]. Studies have shown that macrophage polarization occurs in renal inflammation and plays a decisive part in DN, and secretion of pro-inflammatory mediators by macrophages after activation contributes to deterioration of DN [3]. Macrophages are subdivided into two subpopulations: the classically-activated or M1 phenotype macrophages and the alternatively-activated or M2 macrophages. M1 phenotype macrophages are observed to deliver higher levels of pro-inflammatory cytokines to deal with infections [4, 5]. Macrophages can re-programme their metabolism and differentiate into pro- or anti-inflammatory phenotypes. Inflammatory macrophage activation involves metabolic adaptation towards glycolysis, videlicet, M1 macrophages mainly utilize glycolysis to provide energy [6, 7].

Transforming growth factor β-activated kinase 1-binding protein 1 (TAB1) is a specific protein that interacts with transforming growth factor β-activated kinase 1 (TAK1). TAB1/TAK1 can activate nuclear factor κB (NF-κB) in high glucose (HG)-induced BMMs, regulating macrophage activation [8–12]. Studies suggest that activation of the NF-κB signaling pathway upregulates HIF-1α activity and enhances glycolytic metabolism in an autonomous fashion, HIF-1α may be considered as a novel therapeutic target for DN [13–16]. HIF-1α has been reported to be closely involved in energy metabolism and it increases the activity of glycolytic enzymes such as hexokinase-1 (HK1), 6-phosphofructo-2-kinase/fructose-2, 6-biphosphatase 3 (PFKFB3) and lactate dehydrogenase A (LDHA) [17, 18]. Therefore, TAB1 may be involved in macrophage glycolysis and activation through NF-κB/HIF-1α, which is a principal link in the genesis and evolution of DN.

In this study, we used siRNA and lentiviral vector technology to knock down TAB1 in HG-induced macrophages and STZ-induced diabetic mice, then evaluated glycolysis, polarization of macrophages and levels of inflammation, thereby exploring the relationship between the TAB1/NF-κB/HIF-1α signaling pathway and glycolysis in polarization of macrophages in the DN microenvironment.

## Materials and methods

### Materials and reagents

The following materials and reagents were used in this study: mouse fibroblast cell line (L-929) was obtained from the cell bank of the Chinese Academy of Sciences; lipofectamine RNA iMAX transfection and Trizol reagent were obtained from Thermo Scientific (USA); HiScript II Q RT SuperMix for qPCR and AceQ qPCR SYBR Green Master Mix were obtained from Vazyme (Vazyme, Nanjing, China); A3500 Reverse Transcription System was obtained from Promega (Promega, Madison, USA); FITC anti-mouse F4/80, APC anti-mouse/human CD11b, FITC rat IgG2a κ isotype control and APC rat IgG2a κ isotype control were obtained from BioLegend (San Diego, California, USA); iNOS Monoclonal Antibody (CXNFT), APC were from eBioscience™ from Thermo Fisher Scientific (USA), anti-NF-κB p65 antibody and anti-NF-κB p65 antibody(ChIP) was obtained from Cell Signaling Technology (Beverly, MA, USA); anti HIF-1α, anti-HK1, anti-TAB1 and anti-inducible nitric oxide synthase (iNOS) were obtained from Abcam Biotechnology (Cambridge, UK); anti-PFKFB3, anti-LDHA and anti-P-P65 were from Wuhan Sanying Biotechnology Inc (Sanying, Wuhan, China); anti-CD68 and anti-TNF-α were from Arigo Biolaboratories Corp (Arigo, Taiwan, China); anti-IL-1β from ABclonal Technology (Wuhan, China) and ELISA kits for mouse IL-1β, MCP-1 and TNF-α were obtained from R&D (USA) and MULTI SCIENCES(Hangzhou, China). Glucose absorption kits were obtained from Biovision (USA), Lactic Acid assay kit from Nanjing jiancheng (Nanjing, China) and TAB1-siRNA from Ribobio (Guangzhou, China). TAB1 lentivirus was obtained from Genepharma (Shanghai, China). Immunohistochemical double-stain kit was obtained from Maxin (Fuzhou, China). Mouse microalbumin ELISA kits were from Abcam Biotechnology. Chromatin IP Kit were from Cell Signaling Technology (USA).

### Isolation and culture of BMMs

BMMs were isolated from C57BL/6 J mice (6–8 weeks, male). Mice were killed with ether and bone marrow collected from tibias and femurs. Cells were washed in cold phosphate buffered saline (PBS) + 2% heat-inactivated fetal calf serum (FBS) and then centrifuged at 500 g for 5 min. Cell pellets were resuspended in red blood cell lysis buffer and washed again. Resuspended cells were maintained in DMEM medium supplemented with 15% L-929 cell supernatant, 10% FBS and 1% penicillin/streptomycin. They were cultured at 37° C and 5% CO_2_ and medium was replaced on day 3. The adherent cells were BMMs and were used for subsequent experiments on day 7.

### Flow cytometry (FCM) analyses

For surface marker analysis, BMMs in PBS were blocked with anti-CD16/CD32 to prevent non-specific binding and then washed in PBS and centrifuged for 10 min, the supernatant was decanted and 0.5 ml PBS/tube containing FITC-conjugated anti-mouse F4/80 and APC-conjugated anti-mouse CD11b was added—the samples were incubated in the dark at room temperature for 30 min. For intracellular cytokine staining, the cells were fixed (4% paraformaldehyde) and permeabilized (3% triton × 100), after blocked with anti-CD16/CD32, stained with APC-conjugated iNOS Monoclonal Antibody and FITC-conjugated anti-mouse F4/80. The macrophages were then washed. Finally, 1 ml of PBS was added to the resuspended macrophages, which were analyzed with a BD FACS Calibur flow cytometer.

### Transfection with TAB1 siRNA

Following the manufacturer’s instructions, TAB1 siRNA and the negative control (NC) were transfected into BMMs by lipofectamine RNA iMAX transfection for 24 h and were selected for the optimal transfection band by Western blot. The sequences of TAB1 siRNA were: (1) 5′-GAGGAACTTTGGCTATCCA-3′; (2) 5′-GTGGATGGGTTACAGGTTA-3′; (3) 5-GGATTACAAGGTCAAATAT-3′.

### Experimental groups

First, the stimulation time for d-glucose was optimized. According to previous work of our group, we used 30 mmol/L glucose as a stimulating factor. The BMMs were grouped into: (1) mannitol control group (D-mannitol), (2) negative control siRNA (control siRNA), (3) blank control (control), (4) hyperglycemic stimulation (HG), (5) TAB1 siRNA (TAB1 siRNA), (6) TAB1 siRNA HG stimulation group (TAB1 siRNA + HG).

### ELISA

The levels of Inflammatory cytokines were tested by ELISA. BMMs were treated with TAB1 siRNA or not for 24 h and then stimulated with HG for 24 h; the kidney tissue of each mice was cuting to pieces and then ultrasonically broken with PBS. Supernatants and tissue homogenate were assayed following the manufacturer’s instructions, OD values were measured at 450 nm and actual concentrations calculated using the standard curves.

### Lactic acid production and glucose absorption

BMMs were pre-treated with TAB1 siRNA or not for 24 h, then exposed to HG for 24 h. Their lactic acid production and glucose absorption were detected using the lactic acid production kit and glucose absorption kit.

### Confocal microscopy analysis

BMMs were fixed using paraformaldehyde (4%) at room temperature for 30 min. 5% bovine serum albumin + 0.2% Triton X-100 was used to block for 2 h and BMMs were then incubated with specific primary antibodies at 4 °C overnight. FITC anti-mouse F4/80 and corresponding fluorescent secondary antibody were added in the dark at 37 °C. After washing three times with PBS, the nuclei were stained with 40, 60-dimidyl-2-phenylindole (DAPI) for 10 min and observed under a Leica TCS SP5 laser confocal microscope. The semi-quantitative analysis of laser confocal was performed by ImageJ.

### Western blotting

Renal samples and BMMs were treated with Radio-Immunoprecipitation Assay and phosphatase inhibitor mixed buffer. Proteins were separated by SDS-PAGE electrophoresis and transferred to polyvinylidene difluoride (PVDF) or nitrocellulose (NC) membranes. These were incubated overnight at 4 °C with specific primary antibodies and then stained with horseradish peroxidase-labeled secondary antibody. Detection was performed with enhanced chemiluminescence (ECL) reagent and visualized with the chemiluminescence GE 600 system. Results were expressed as relative ratios and quantified by Image J.

### RNA extraction and qRT- PCR

Following manufacturer’s instructions, total RNA was extracted from the BMMs using Trizol reagent. 1 μg RNA was reverse transcribed to cDNA using the Reverse Transcription System. The cDNA was amplified by real-time PCR with AceQ qPCR SYBR Green Master Mix. Primers to detect mRNA were as follows: GAPDH, as the internal control: Forward primer: 5′-ACCCCAGCAAGGACACTGAGCAAG-3′, Reverse primer: 5′-GGCCCCTCCTGTTATTATGGGGGT-3′; HK1: Forward primer: 5′-TGCCATGCGGCTCTCTGATG-3′, Reverse primer: 5′-CTTGACGGAGGCCGTTGGGTT-3′; PFKFB3: Forward primer: 5′-AGCCAGCTACCAGCCTCTTG-3′, Reverse primer: 5′-AATTCGGCTCTGGATGTGGT-3′; LDHA: Forward primer: 5′-CAAAGACTACTGTGTAACTGCGA-3′, Reverse primer: 5′-TGGACTGTACTTGACAATGTTGG-3′; MCP-1: Forward primer: 5′-TTGACCCGTAAATCTGAAGCTAAT-3′, Reverse primer: 5′-TCACAGTCCGAGTCACACTAGTTCAC-3′; IL-1β: Forward primer: 5′- GCCTCGTGCTGTCGGACCCATAT-3′, Reverse primer: 5′-TCCTTTGAGGCCCAAGGCCACA-3′. The relative mRNA levels were calculated by the 2-△△Ct method with values normalized to the GAPDH reference gene.

### Mice

The study was performed according to the international, national and institutional rules concerning animal experiments and the Animal Ethics Committees of the Faculty of Anhui Medical University approved all experimental protocols, in accordance with “Principles of Laboratory Animal Care and Use in Research” (Ministry of Health, Beijing, China). 24 C57BL/6 J littermate male mice (6–8 weeks) were obtained from the Model Animal Research Center of Nanjing University and were maintained in Specific Pathogen Free conditions at 24 °C and 60% humidity, light and dark for 12 h each day. Mice were housed in separate cages and were free to eat and drink. Mice were given STZ (Sigma Chemical Co., St. Louis, MO, USA) at 50 mg/kg body weight daily for 5 consecutive days to establish diabetes. Animal groups (*n* = 6 each group) were: C (control), STZ, STZ + Negative control (NC) lentivirus, STZ + TAB1 lentivirus. TAB1 lentivirus (5′-GTGGATGGGTTACAGGTTA-3′) was administered weekly to the STZ + TAB1 lentivirus group mice through the tail vein at a dose of 1 × 10^9^, 50 µl. NC lentivirus was administered at the same dose as the STZ + NC lentivirus group. Other groups of mice were injected with the same amount of normal saline via the tail vein (C group and STZ group).

### Physical and biochemical analyses

Body and kidney weight were measured and blood glucose was assessed when the mice were sacrificed after 12 weeks. Twenty-four-hour urine was collected through metabolic cages and urine volume calculated. Urinary albumin levels were assayed using a mouse microalbumin ELISA kit according to the manufacturer’s instructions.

### Histology and Immunohistochemistry

All mice were sacrificed after 12 weeks. Then, 4% paraformaldehyde-fixed and paraffin-embedded fresh renal tissue sections were cut into sections of around 3 µm thickness. After deparaffinization, periodic acid–Schiff (PAS) staining was used to assess pathological damage to the kidney. The glomerular mesangial expansion index and the tubulointerstitial injury index were evaluated and graded using 10 randomly selected visual fields.

After antigen retrieval, tissue sections were blocked with serum for 30 min at 37 °C and then incubated with primary antibody (overnight at 4 °C). After incubation with secondary antibody for 1 h at 37 °C, they were stained with 3, 3-diaminobenzidine and hematoxylin. Staining for TAB1, CD68, TNF-α, IL-1β, HK1, PFKFB3 and LDHA was detected using the Image J System. According to the double immunohistochemical staining kit instructions, incubation and staining with the two primary antibodies was performed separately and the results observed under a standard microscope.

### Chromatin immunoprecipitation

Chromatin immunoprecipitation (ChIP) assay was performed using the e SimpleChIP^®^ Enzymatic Chromatin IP Kit (Magnetic Beads) #9005 (Cell Signaling Technology, USA) according to the manufacturer’s instructions. Chromatin was precipitated with NF-κB p65 antibodies and samples were analyzed by PCR. The murine HIF-1α promoters were amplified with the primer pairs: Forward primer: 5′-TGTGGCTTCCAACTGGGTGTT-3′, Reverse primer: 5′-AGGGTCAGCAGAATACCCCT-3'.

### Statistical analysis

Statistical analysis was conducted using SPSS software version 17.0 (SPSS Inc. Chicago, IL, USA). All data were analyzed by one-way analysis of variance (ANOVA) and presented as mean ± standard deviation (SD). The difference between groups was tested by least significant difference (LSD) and the Levene method was used for homogeneity test of variance. *p* values < 0.05 were considered to be statistically significant.

## Results

### Determination of purity and maturity of BMMs

**Fig. 1:**
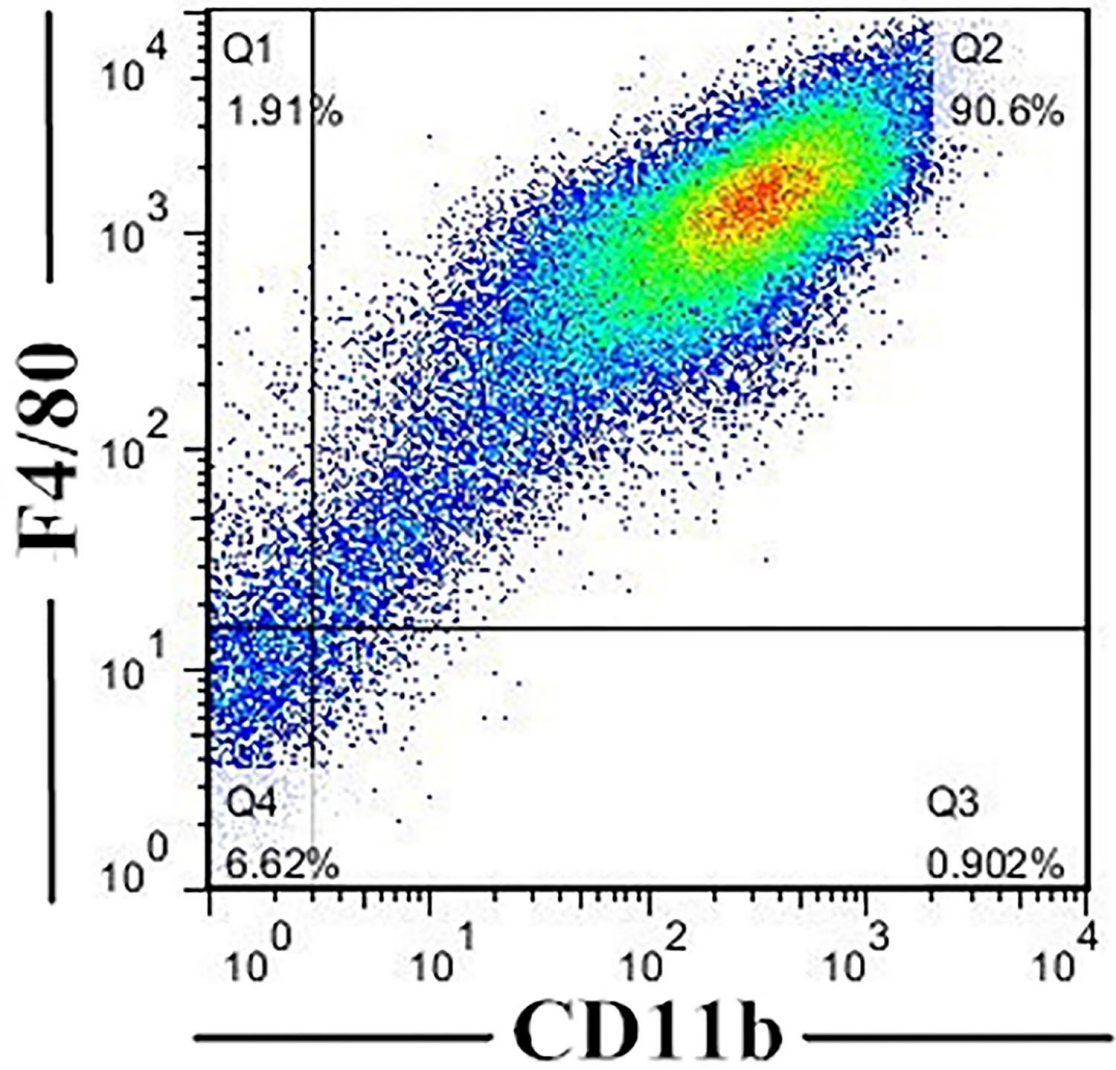
Identification of BMMs. Mature BMMS were defined as F4/80^+^ CD11b^+^ cells by flow cytometry. 90.6% of them were gated as F4/80 and CD11b double positive cells

The development of BMMs was assessed by flow cytometry (Fig. 1). F4/80 and CD11b are well-known markers for macrophages. Mature BMMs were defined as F4/80 and CD11b positive cells. High purity and maturity of BMMs could be obtained on day 7, when 90.6% were F4/80^+^ and CD11b^+^.

### Effects of HG on the protein level of HK1, PFKFB3, LDHA, TAB1, NF-κB p65 and HIF-1α at different time points, and the interaction of NF-κB p65 and HIF-1α

**Fig. 2:**
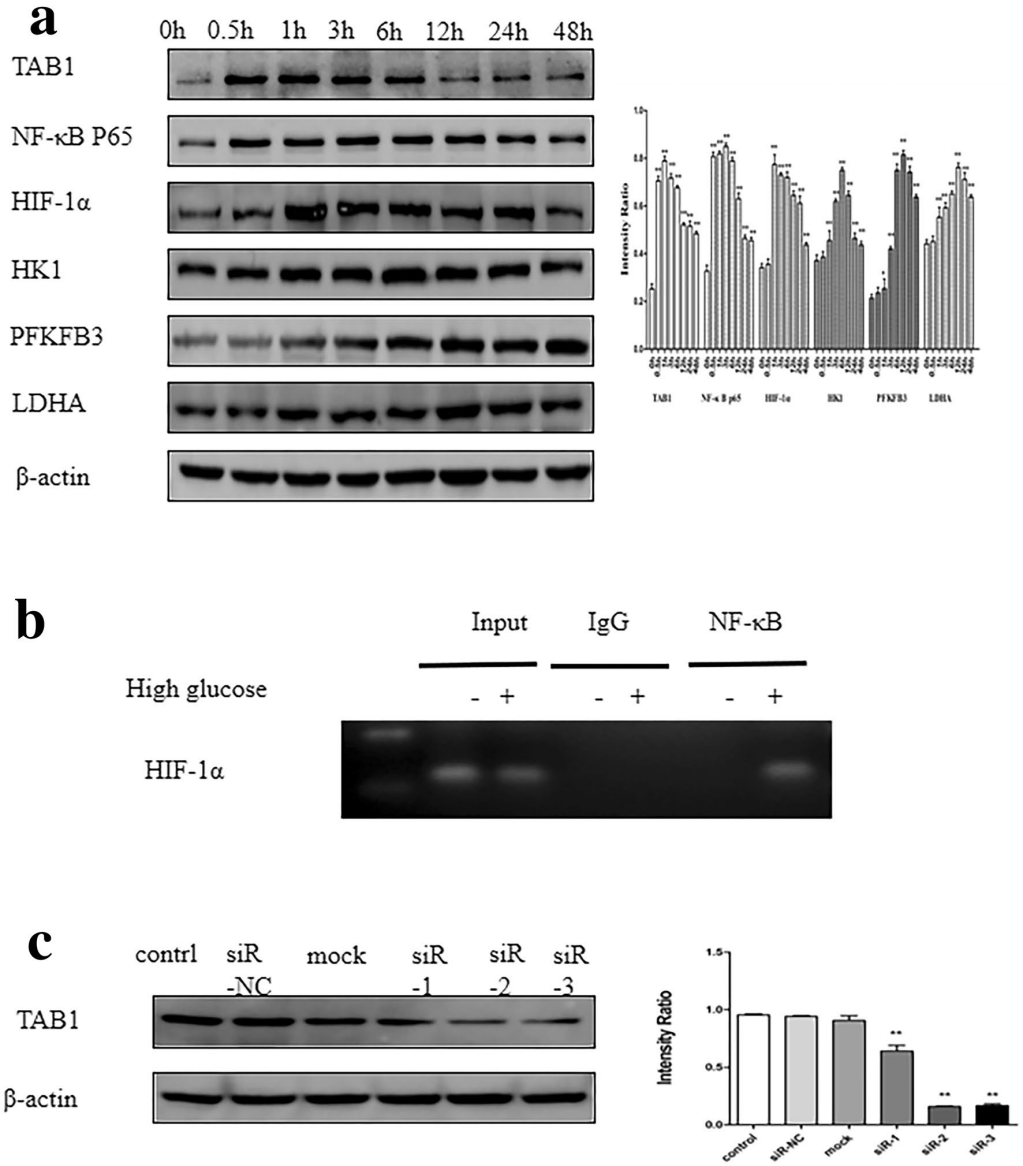
More glycolytic enzymes and TAB1/NF-κB p65/HIF-1α are expressed in HG treated macrophages. **a** Western blotting analysis of HK1, PFKFB3, LDHA, TAB1, NF-κB p65 and HIF-1α in activated BMMs and densitometric analysis over time. BMMs were pre-treated with HG for 0, 0.5, 1, 3, 6, 12, 24 and 48 h, *n* = 3. Values are means ± SD, *p < 0.05, **p < 0.01 vs 0 h. **b** Binding of NF-κB p65 to HIF-1α by ChIP assay. **c** Effect of TAB1 siRNA transfection on intracellular TAB1 protein expression. Western blotting was used to detect protein expression of TAB1, *n* = 3. Values are means ± SD, **p < 0.01 vs control

Western blotting analyses revealed novel upregulation of TAB1 protein levels at 0.5 h, which peaked at 1 h after treatment with 30 mmol/L HG (Fig. 2a, *p* < 0.01). NF-κB p65 began to over-express at 0.5 h and peaked at 3 h. One hour after HG stimulation, the protein level of HIF-1α began to increase and reached a peak before gradually decreasing. Glycolysis enzymes, such as HK1, PFKFB3 and LDHA increased after an hour, with peaks at 6, 12, and 12 h, respectively. ChIP assay detected the binding of NF-κB p65 on the HIF-1α promoter region in high glucose -stimulated macrophages (Fig. 2b).

### TAB1 siRNA decreased the expression of TAB1 in BMMs

The effect of three bands of TAB1 siRNA on inhibition of TAB1 protein expression was examined 24 h after transfection. As shown in Fig. 2c, the results suggested that three bands of TAB1 siRNA could downregulate the protein level of TAB1 (*p* < 0.01). siR-2 could significantly decrease the level of TAB1 (*p* < 0.01)—thus siR-2 was selected for subsequent experiments.

### TAB1 siRNA inhibited HG-induced BMM differentiation into a pro-inflammatory phenotype

**Fig. 3:**
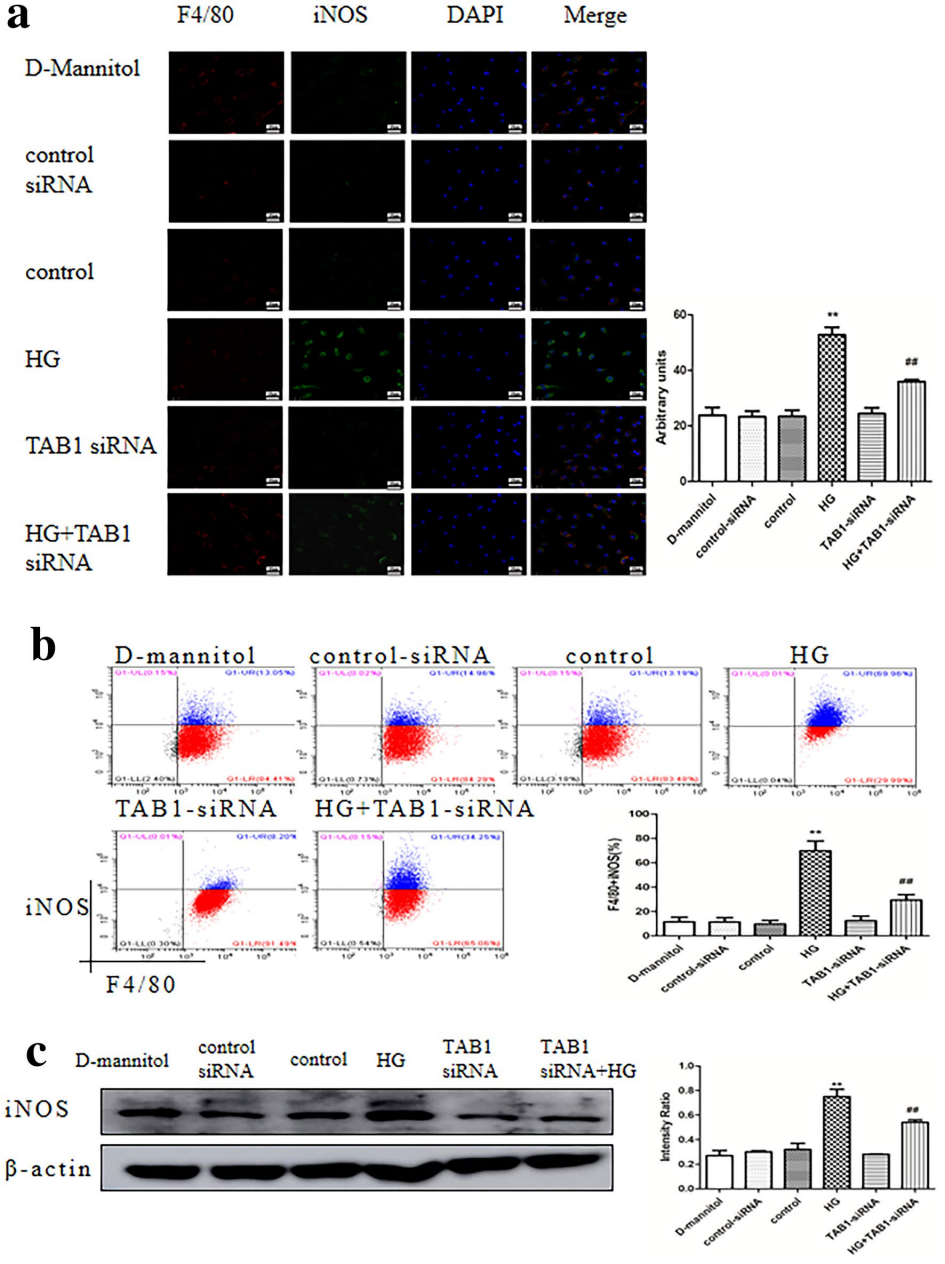
Effects of TAB1 siRNA on the expression of M1 macrophage markers in HG-induced macrophages. **a** Coexpression of iNOS and F4/80 in HG-induced macrophages evaluated by confocal microscopy. Effects of TAB1 siRNA on the activation of macrophages after HG treatment were evaluated by confocal microscopy. Scale bars = 25 μm. **b** Flow cytometry assay was used to detect F4/80 and iNOS in macrophages, *n* = 3. Values are means ± SD. **p < 0.01 vs control, ^##^*p* < 0.01 vs HG. **c** Western blotting used to detect the protein expression of iNOS in macrophages. *n* = 3, values are the means ± SD. **p < 0.01 vs control, ^##^*p* < 0.01 vs HG

Laser confocal microscopy revealed that all BMMs expressed F4/80. Control BMMs showed weak iNOS expression and the positivity of iNOS was significantly enhanced in the HG group, which indicated that HG could cause differentiation into a pro-inflammatory M1 phenotype. There was no obvious difference in staining in the TAB1 siRNA group. The green fluorescence intensity of iNOS was weakened in the TAB1 siRNA + HG group when compared with the HG-induced macrophages, suggesting that TAB1 silencing could attenuate BMM differentiation into M1 macrophages (Fig. 3a). Flow cytometry results indicated that the proportion of F4/80^+^ and iNOS^+^ macrophages increased significantly in HG and was significantly lower in the HG + TAB1 siRNA group than in HG (Fig. 3b). This phenomenon is consistent with the western blotting expression of iNOS (Fig. 3c).

### Detection of MCP-1 and IL-1β levels in supernatants by ELISA and macrophage mRNA levels.

**Fig. 4:**
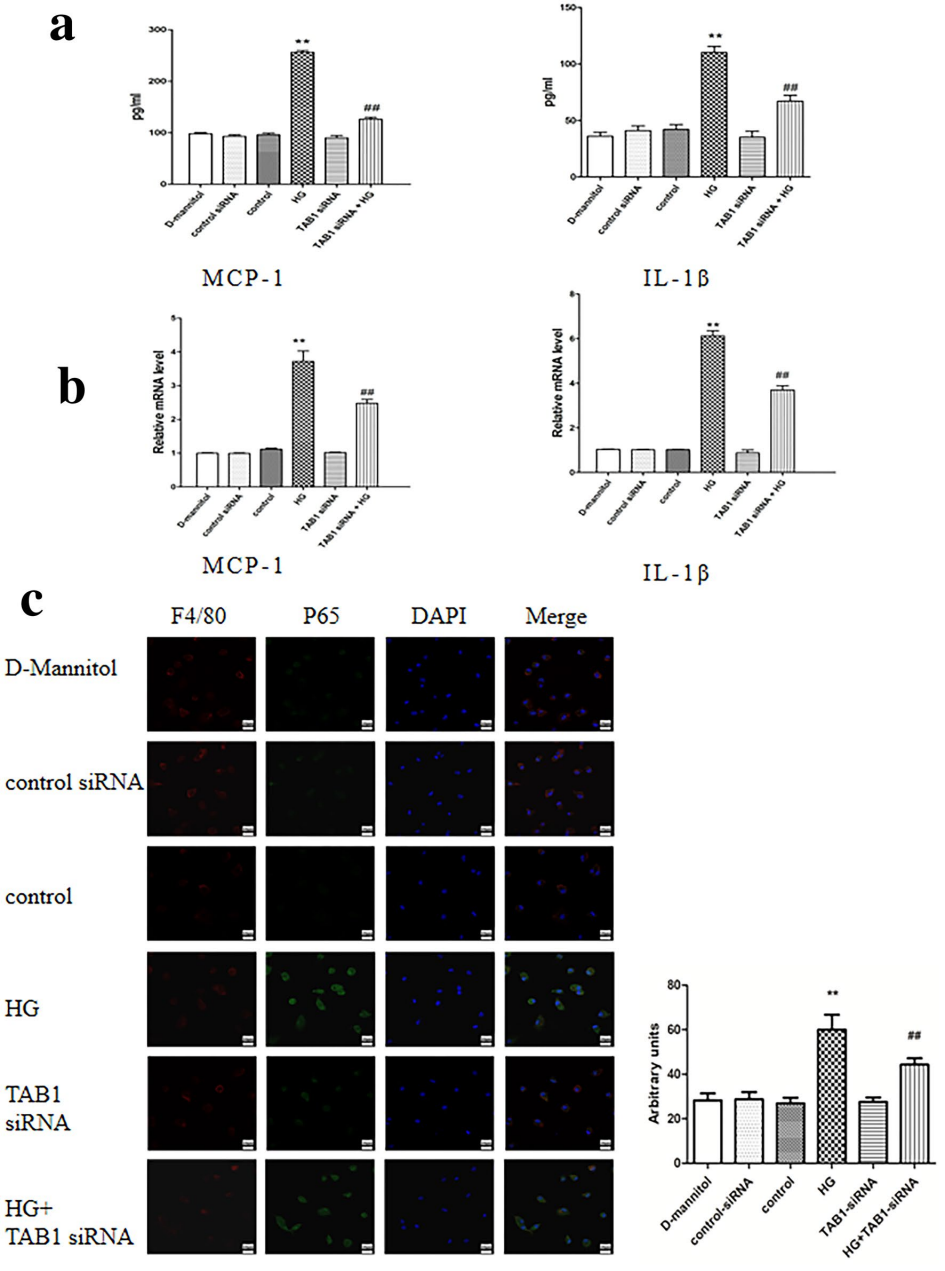
Effects of TAB1 siRNA on the expression of inflammation in HG-induced macrophages. **a** ELISA was used to detect MCP-1 and IL-1β secreted into the cell supernatant, *n* = 3. Values are means ± SD. **p < 0.01 vs control, ^##^*p* < 0.01 vs HG. **b** qRT-PCR detection of mRNA expression of MCP-1 and IL-1β in each group, *n* = 3. Values are means ± SD. **p < 0.01 vs control, ^##^*p* < 0.01 vs HG. **c** Effects of TAB1 siRNA on NF-κB p65 nuclear translocation in HG-induced macrophages—coexpression of F4/80 and NF-κB P65 in HG-induced macrophages was evaluated by confocal microscopy

HG significantly enhanced the expression of IL-1β and MCP-1 in the BMM supernatant compared to control (*p* < 0.01). Furthermore, compared with the HG group, the IL-1β and MCP-1 levels in the BMM supernatant of the TAB1 siRNA + HG group were significantly reduced (*p* < 0.01; Fig. 4a). The results for mRNA were coincident with these data (Fig. 4b).

### Effects of TAB1 siRNA on nuclear translocation of p65

The results of nuclear translocation of NF-κB observed by confocal microscopy indicated that P65 labeled with green fluorescence was spread over the cytoplasmic region rather than the nuclear region in the control group. By contrast, green fluorescence was primarily spread over the nuclear area in HG group BMMs. TAB1 siRNA could significantly inhibit the presence of green fluorescence in the nucleus (Fig. 4c).

### Changes in glycolytic enzymes HK1, PFKFB3 and LDHA under different treatments by confocal laser scanning

**Fig. 5:**
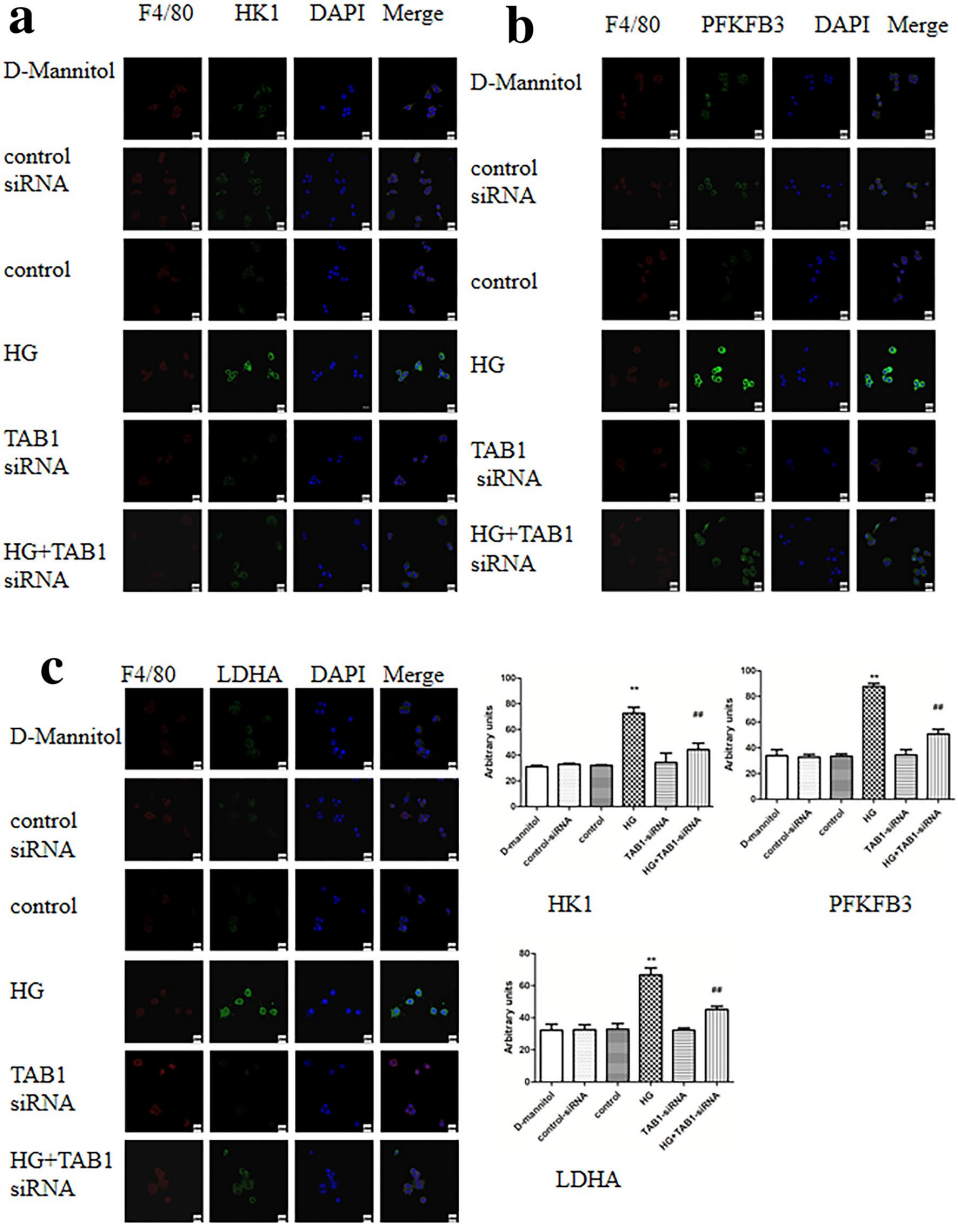
**a**–**c** Effects of TAB1 siRNA on the glycolysis of BMMs—coexpression of F4/80 and HK1/PFKFB3/LDHA in BMMs was evaluated by confocal microscopy. Scale bars = 20 μm

**Fig. 6:**
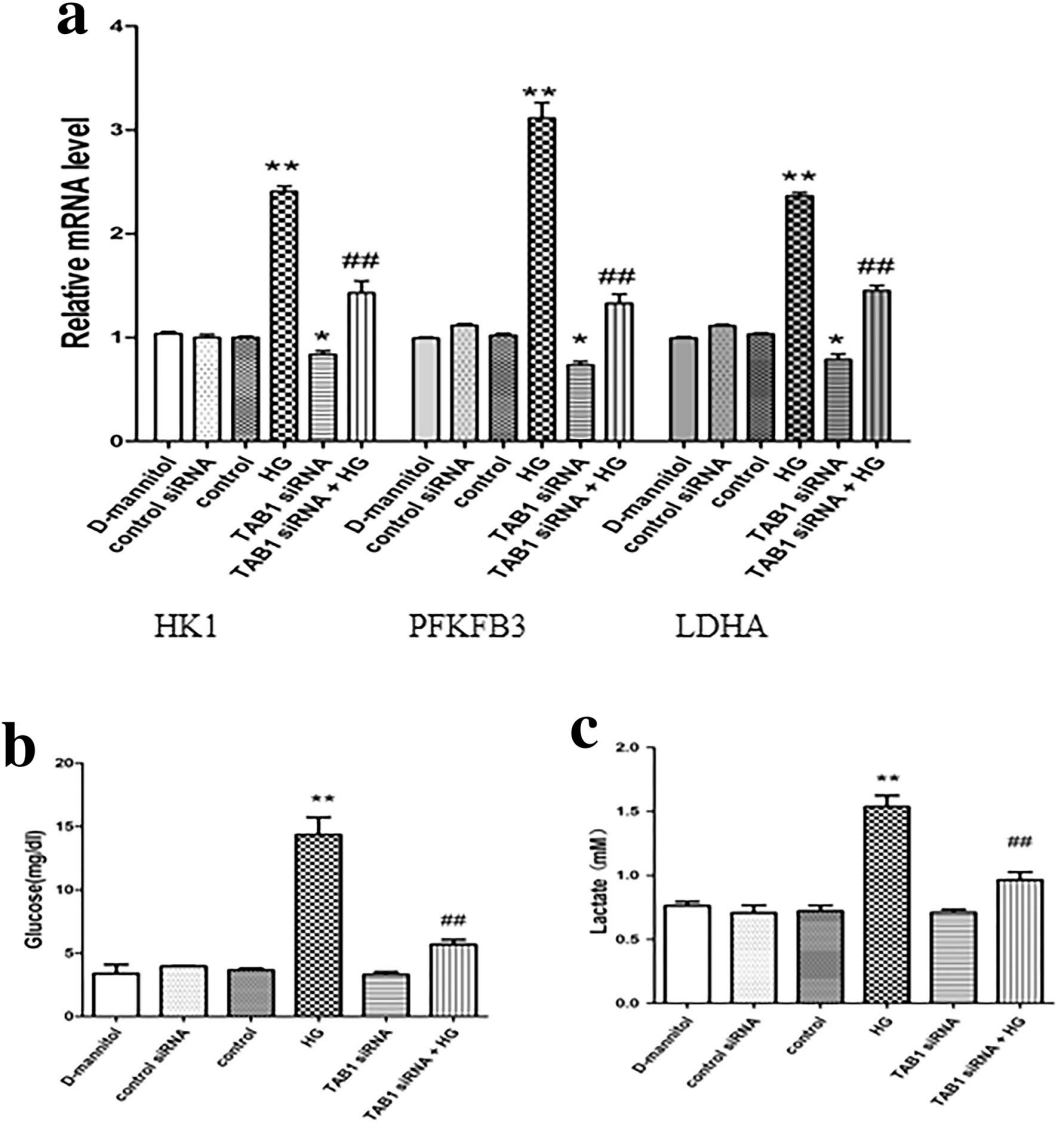
Effects of TAB1 siRNA on the glycolysis of HG-induced BMMs. **a** qRT-PCR detection of changes in the expression of HK1, PFKFB3 and LDHA in each group, *n* = 3. Values are means ± SD, **p < 0.01 vs control, ^##^*p* < 0.01 vs HG. Changes in glucose absorption (**b**) and glucose absorption (**c**) in each group, *n* = 3. Values are means ± SD, **p < 0.01 vs control, ^##^*p* < 0.01 vs HG

Nuclei of BMMs was stained in blue. HK1, PFKFB3 and LDHA were stained green and F4/80 in red. It can be seen from Fig. 5a–c that in the HG group, stronger staining for HK1, PFKFB3 and LDHA were observed, in stark contrast to the control group. However, HG-induced BMMs with TAB1 siRNA showed a reduction in HK1, PFKFB3 and LDHA staining. Of note, the mRNA expression of HK1, PFKFB3 and LDHA in the TAB1 siRNA group was significantly lower than that in the control group (*p* < 0.05) and their expression in the HG group was significantly increased (*p* < 0.01). Compared with the HG group, mRNA expression of HK1, PFKFB3 and LDHA in the TAB1 siRNA + HG group was significantly decreased (*p* < 0.01; Fig. 6a).

### Glycolysis function determined by lactate assay kit and glucose absorption kit

Cell culture medium and cells were collected and the lactic acid production and glucose uptake of BMMs with different treatments were detected to evaluate changes of glycolytic function in macrophages in each group. Compared with control, lactic acid content in the supernatant of the HG-induced BMMs was greatly increased (*p* < 0.01). Lactic acid content of the TAB1 siRNA + HG group statistically decreased when compared with the HG group. Compared with the control, the glucose uptake capacity in the HG group was markedly increased (*p* < 0.01), while glucose uptake capacity in the TAB1 siRNA group was not obviously changed and the difference was not statistically significant. When compared with the HG group BMMs, glucose uptake capacity in the TAB1 siRNA + HG group statistically decreased (*p* < 0.01; Fig. 6b-c). Our results indicate that HG-induced macrophages have upregulated glycolysis and glucose uptake and increased lactic acid content. TAB1 siRNA inhibits HG-induced glycolysis, so TAB1 may be involved in the regulation of glycolysis of M1 macrophages in an HG environment.

### Expression of HK1, PFKFB3, LDHA, TAB1, NF-κB p65 and HIF-1α in HG-induced BMMs

**Fig. 7:**
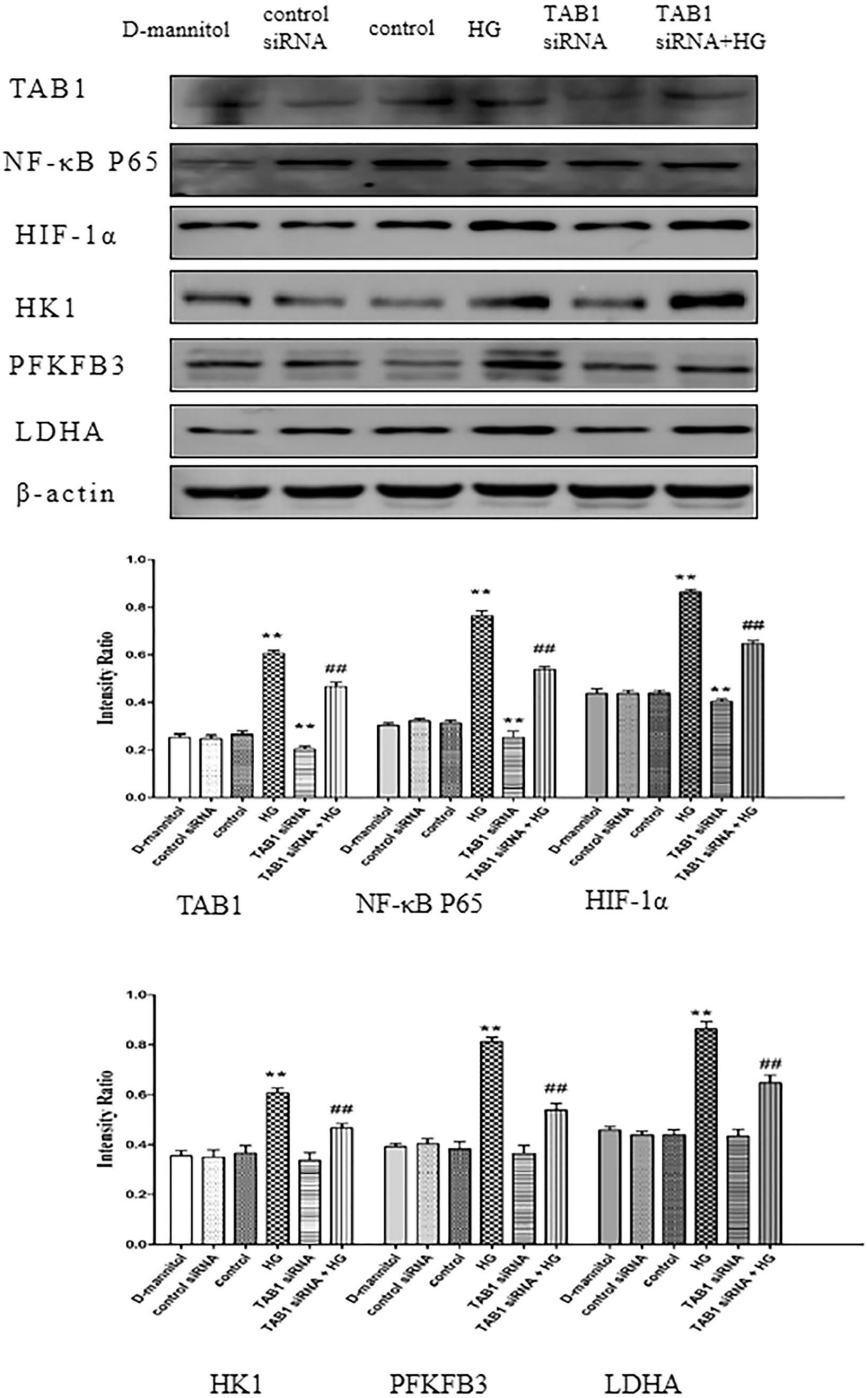
Western blotting used to detect the protein expression of HK1, PFKFB3, LDHA, TAB1, NF-κB p65 and HIF-1α in macrophages. *n* = 3, values are the means ± SD. **p < 0.01 vs control, ^##^*p* < 0.01 vs HG

In Fig. 7, the results of Western blotting showed that the expression of HK1, PFKFB3, LDHA, TAB1, NF-κB p65 and HIF-1α were increased significantly in HG-induced BMMs (*p* < 0.01). Moreover, in the TAB1 siRNA + HG group, the HK1, PFKFB3, LDHA, TAB1, NF-κB p65 and HIF-1α levels of BMMs were reduced compared with those in HG-induced BMMs (*p* < 0.01).

### TAB1 lentivirus decreased expression of TAB1 and effects of TAB1 lentivirus on clinical parameters of DN

**Fig. 8:**
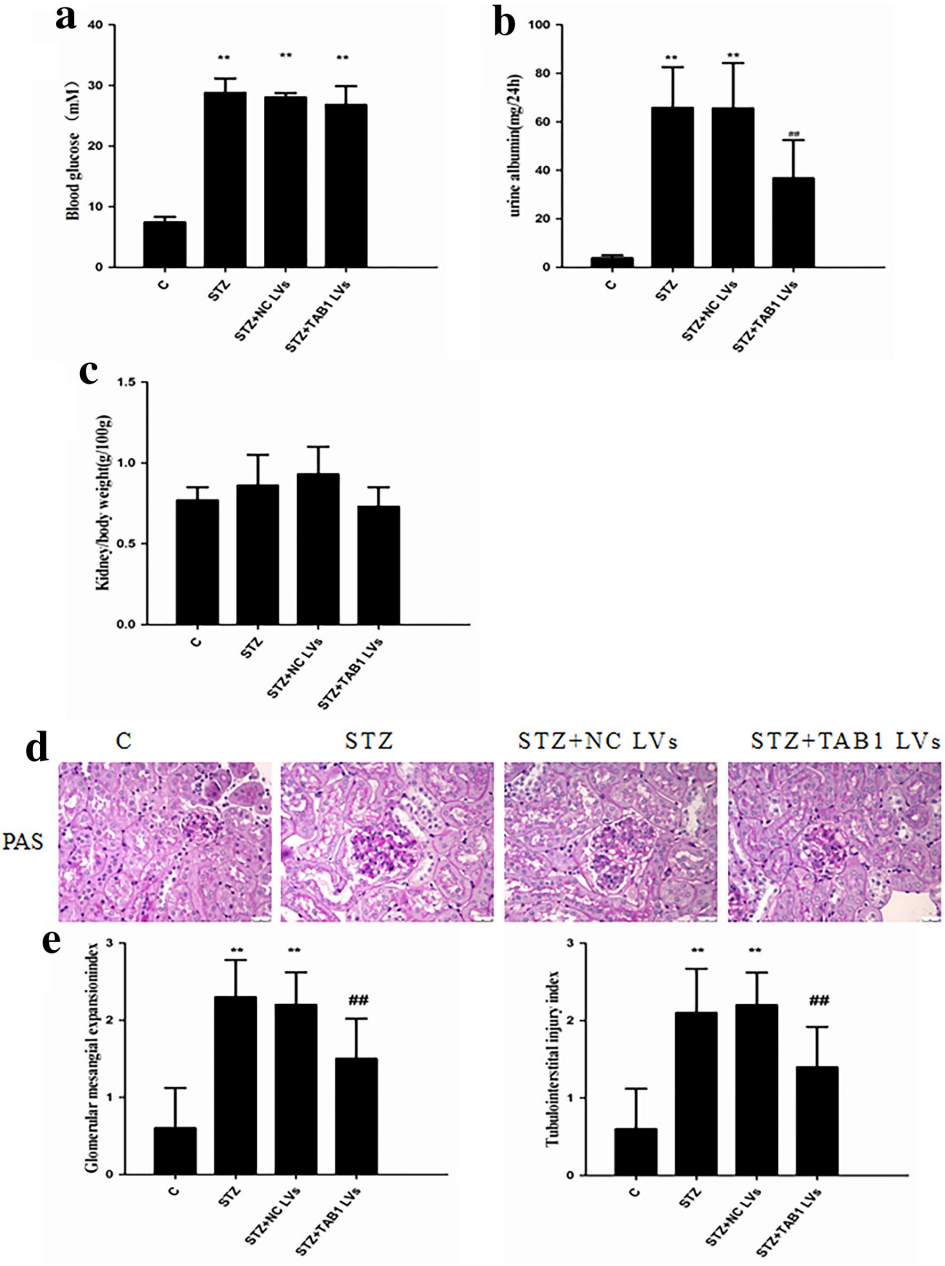
Effect of TAB1 lentivirus (LV) on the clinical and pathological manifestations of DN. **a** Blood glucose levels, **b** Urine albumin excretion, **c** Kidney /body weight, **d**, **e** Histological observations of kidney sections stained with PAS. Scale bars = 20 μm, *n* = 6, values are means ± SD. **p < 0.01 vs control, ^##^*p* < 0.01 vs STZ and STZ + NC lentivirus (LV)

Mice treated with streptozotocin had significantly higher blood glucose than normal mice (> 16.7 mmol/L). However, TAB1 lentivirus did not reduce blood glucose (Fig. 8a). Compared with normal mice, STZ-injected mice showed increased 24-h urine albumin excretion. Interestingly, TAB1 lentivirus weakened urine albumin excretion of the diabetic group. However, although the trends were similar, kidney/body weight levels in these mice showed no statistical differences (Fig. 8b, c).

**Fig. 9:**
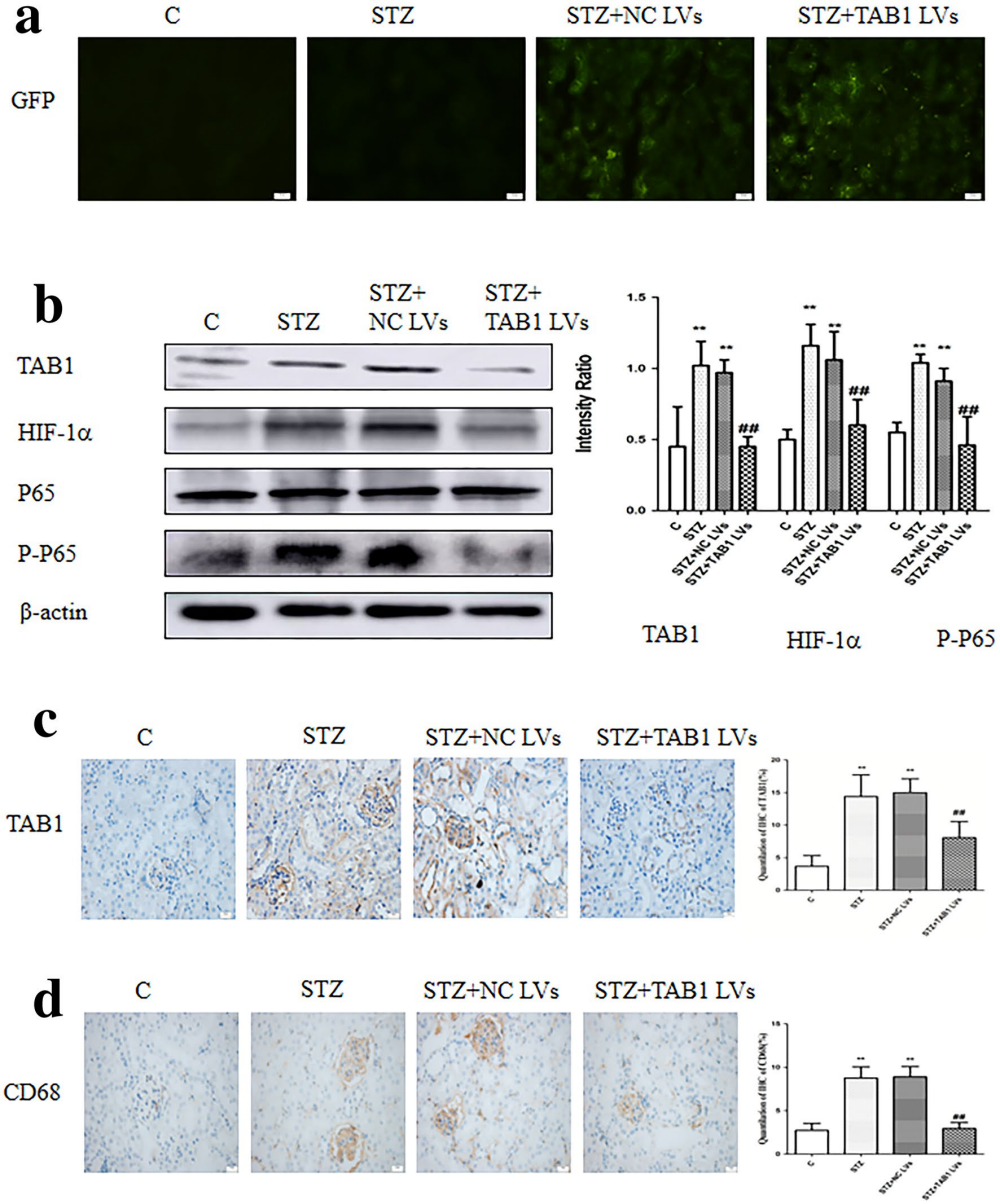
Effects of TAB1 LV on the expression of TAB1, downstream signaling pathway molecules and infiltration of macrophages and in different groups. **a** Expression of GFP fluorescence from lentivirus in diabetic mouse kidney evaluated by fluorescence microscopy. **b** Renal tissue analyzed by Western blotting to measure the expression of TAB1 and molecules of its downstream signaling pathway, including HIF-1α, NF-κB p-p65, and NF-κB p65. **c**, **d** Immunohistochemistry (IHC) analysis of TAB1 and CD68, Scale bars = 20 μm, *n* = 6. Values are means ± SD. **p < 0.01 vs control, ^##^*p* < 0.01 vs STZ and STZ + NC LVs

Fluorescence microscopy revealed that compared to no treatment, lentivirus-transfected cells were stained green by green fluorescent protein (GFP; Fig. 9a). Results from Western blotting showed that renal tissue in the STZ group (STZ, STZ + NC lentivirus) showed remarkably higher TAB1/HIF-1α/NF-κB levels than those in control. Expression of TAB1/HIF-1α/NF-κB in the kidneys of the STZ + TAB1 lentivirus group mice was significantly decreased compared with the other diabetic mice (STZ, STZ + NC lentivirus) (*p* < 0.01). These results were supported by immunohistochemistry, which revealed less positive staining for TAB1 in the STZ + TAB1 lentivirus group (*p* < 0.01; Fig. 9b, c).

### TAB1 lentivirus attenuates pathological manifestations of DN

Microscopic examination of PAS stained sections revealed classic glomerular sclerosis and tubular injury in STZ-diabetic mice. The tubulointerstitial damage index and glomerular mesangial expansion index were statistically significantly increased. TAB1 lentivirus significantly ameliorated these DN pathological manifestations (*p* < 0.01; Fig. 8d, e).

### TAB1 knockdown reversed STZ-induced upregulation of inflammation in vivo

**Fig. 10:**
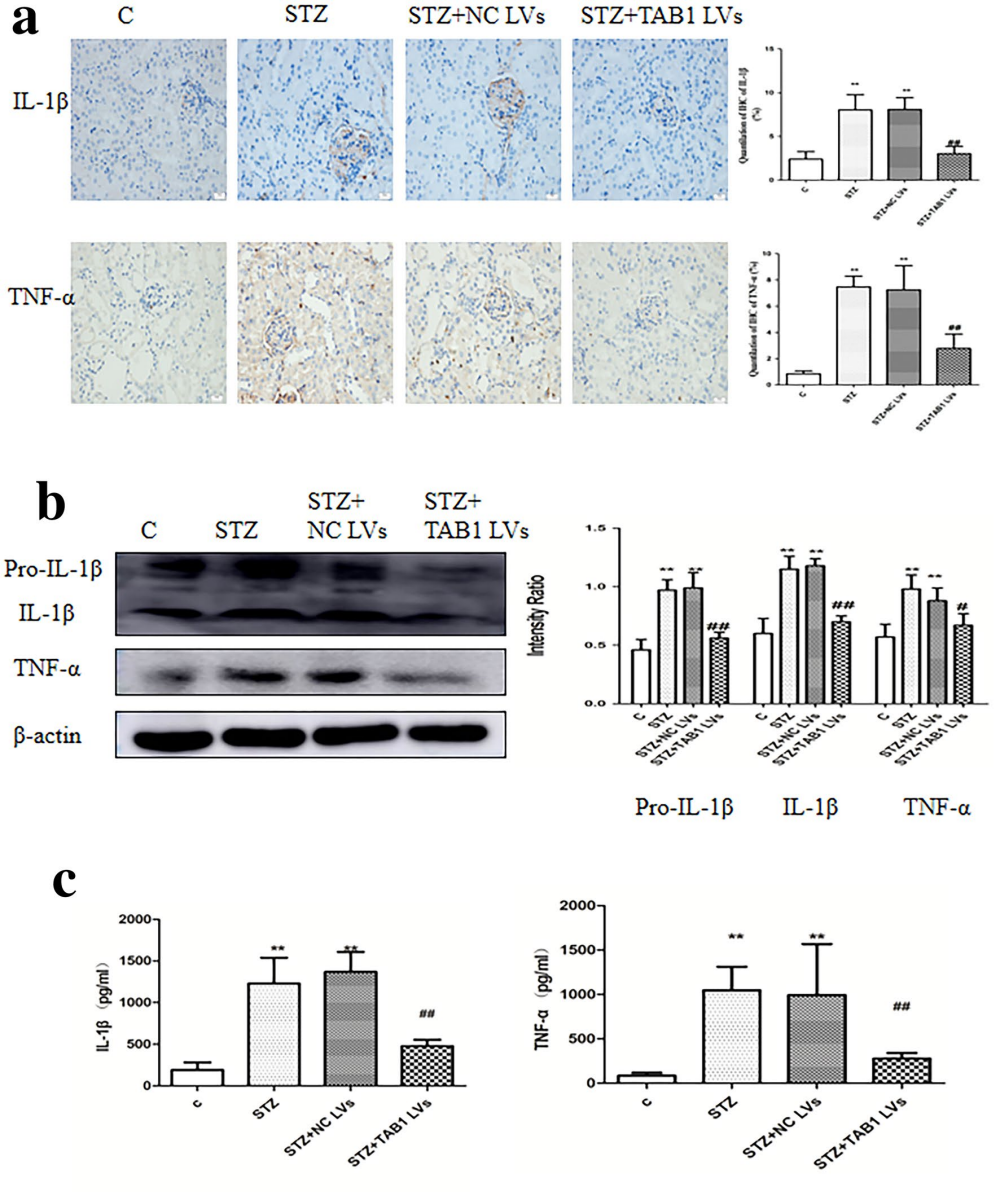
Effects of TAB1 LV on inflammation in different groups. **a** Immunohistochemistry (IHC) analysis of CD68, IL-1β, and TNF-α in the kidney. Scale bars = 20 μm, *n* = 6, values are means ± SD. **p < 0.01 vs control, ^##^*p* < 0.01 vs STZ and STZ + NC LV. **b** Western blotting analysis of IL-1β and TNF-α in the kidney. *n* = 6, values are means ± SD. **p < 0.01 vs control, ^#^*p* < 0.05 vs STZ and STZ + NC LV, ^##^*p* < 0.01 vs STZ and STZ + NC LV. **c** ELISA was used to detect TNF-α and IL-1β in the kidney. *n* = 6, values are means ± SD. **p < 0.01 vs control, ^#^*p* < 0.05 vs STZ and STZ + NC LV, ^##^*p* < 0.01 vs STZ and STZ + NC LV

CD68, a parameter reflecting renal macrophage accumulation, can be considered a readout for the main clinical feature of DN. Immunohistochemical results showed increased CD68 in the renal tissue of diabetic mice. By contrast, TAB1 lentivirus markedly attenuated the upregulation of CD68 induced by STZ (*p* < 0.01; Fig. 9d). Western blotting analysis and ELISA showed that IL-1β and TNF-α were statistically increased in the STZ and STZ + NC lentivirus group compared to control. Furthermore, the STZ + TAB1 lentivirus groups showed distinctly decreased expression. This phenomenon is consistent with the immunohistochemical expression of IL-1β and TNF-α (*p* < 0.01; Fig. 10a–c).

### Changes in glycolytic enzymes HK1, PFKFB3 and LDHA

**Fig. 11:**
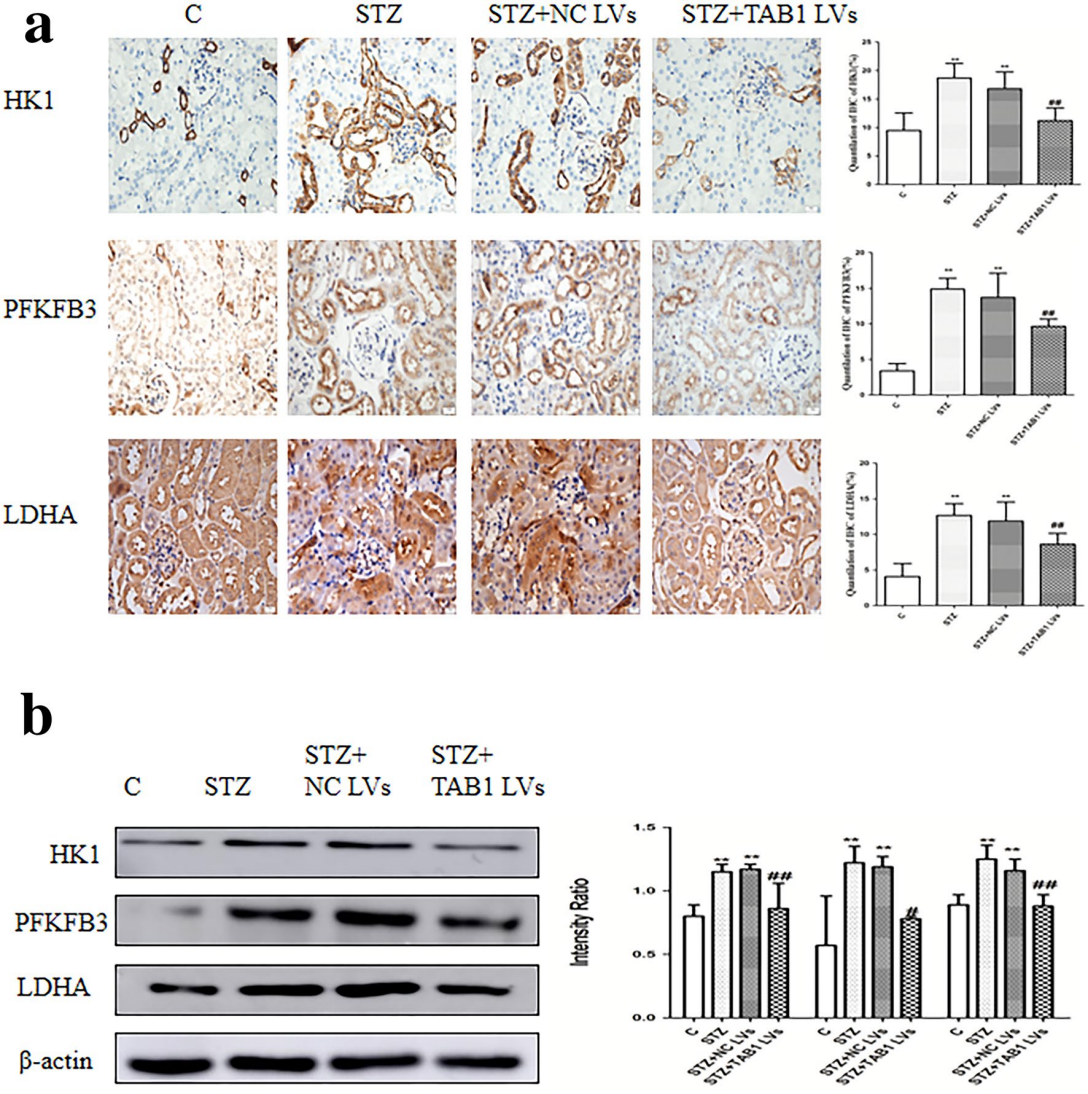
Effects of TAB1 LV on glycolysis in different groups. **a** Immunohistochemical analysis of HK1, PFKFB3 and LDHA in the kidney. Scale bars = 20 μm, *n* = 6, values are means ± SD. **p < 0.01 vs control, ^#^*p* < 0.05 vs STZ and STZ + NC LV, ^##^*p* < 0.01 vs STZ and STZ + NC LV. **b** Western blotting analysis of HK1, PFKFB3 and LDHA in the kidney. *n* = 6, values are means ± SD. **p < 0.01 vs control, ^#^*p* < 0.05 vs STZ and STZ + NC LV, ^##^*p* < 0.01 vs STZ and STZ + NC LV

The expression of HK1, PFKFB3 and LDHA revealed by immunohistochemistry in mouse kidneys were increased in diabetic renal tissue, while TAB1 lentivirus decreased glycolytic enzyme expression significantly. Similarly, Western blotting results indicated that renal tissue of diabetic mice had higher expression of HK1, PFKFB3 and LDHA compared to control and the presence of TAB1 lentivirus prevented this increase (*p* < 0.01; Fig. 11a–b).

### TAB1 lentivirus alters macrophage glycolysis and polarization in kidneys of diabetic mice

**Fig. 12:**
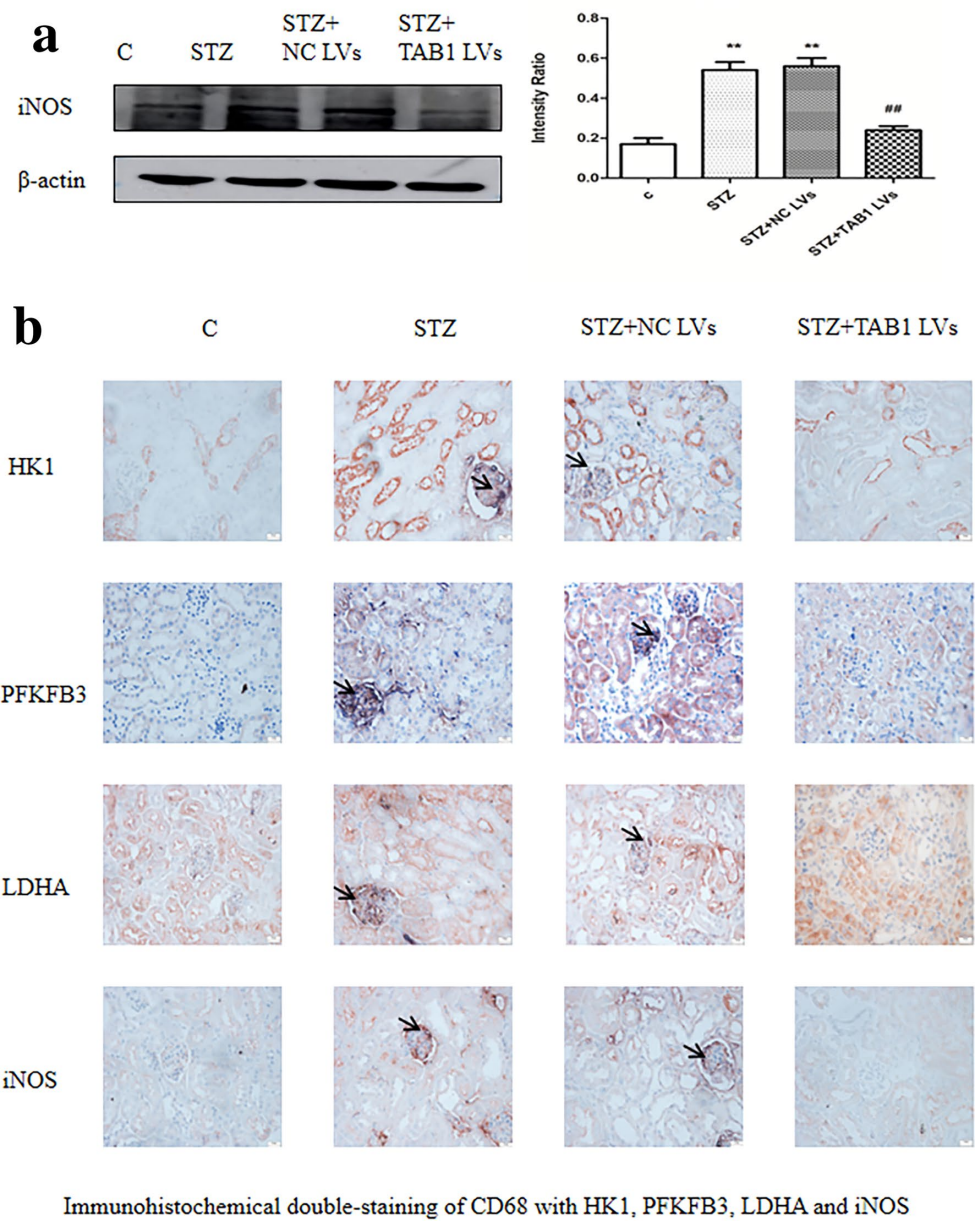
Effects of TAB1 LV on glycolysis and polarization of macrophages. **a** Western blotting analysis of iNOS in the kidney. *n* = 6, values are means ± SD. **p < 0.01 vs control, ^#^*p* < 0.05 vs STZ and STZ + NC LV, ^##^*p* < 0.01 vs STZ and STZ + NC LV. **b** Coexpression of CD68 and HK1/PFKFB3/LDHA/iNOS in mouse kidneys assessed by immunohistochemical double staining. Scale bars = 20 μm

Western blotting results indicated that renal tissue of diabetic mice had higher expression of iNOS compared to control and the presence of TAB1 lentivirus prevented this increase (*p* < 0.01; Fig. 12a). By double immunohistochemical staining**,** HK1, PFKFB3 and LDHA were stained pink, and CD68 in dark blue. In the STZ group and STZ + NC lentivirus, stronger staining for HK1, PFKFB3 and LDHA were observed, in stark contrast to the control group. However, STZ-induced diabetic mice with TAB1 lentivirus showed a reduction in CD68 and HK1, PFKFB3 and LDHA staining. iNOS is an M1 macrophage marker. Compared with controls, the STZ and STZ + NC lentivirus groups had notably enhanced CD68 (pink) and iNOS (dark blue) signals, while knockdown of TAB1 could inhibit the enhancement of these signals (Fig. 12b).

## Discussion

DN is a frequent microvascular complication in diabetes but current treatments, such as blood glucose and blood pressure control and renin–angiotensin–aldosterone system inhibitors, remain suboptimal. Increasingly, research has suggested that inflammation is a critical factor activated by metabolic, hyperglycaemic, AGEs [19] and haemodynamic derangements. Macrophages may be pivotal to this process and unexplained inflammatory cells are known to mediate kidney inflammation [20–22]. Research has shown that macrophage infiltration is closely involved in regulation of serum creatinine, proteinuria, and interstitial fibrosis [23].

Macrophages have two major subtypes: the M1 macrophage (or classically activated) is involved in inflammation by secreting pro-inflammatory cytokines in response to pathogens or other immune stimuli (including but not limited to DN). Conversely, the M2 macrophage exhibits anti-inflammatory properties [24]. In vivo, M1 macrophage polarization and proinflammatory cytokine expression increase, and M2 macrophage polarization and anti-inflammatory cytokine expression decrease in DN mice. The imbalance of M1/M2 macrophages reflects the pathogenesis of DN. In vitro, DN microenvironmental products, such as high glucose and AGEs tend to differentiate macrophages into the M1 phenotype, secreting more iNOS and pro-inflammatory cytokines including IL-1β, TNF-α and MCP-1 [25, 26]. Our study suggests that expression of iNOS in HG-induced BMMs is markedly enhanced. The mRNA expression and secretion of inflammatory cytokines MCP-1 and IL-1β, were significantly elevated in the HG group. The result that HG induces polarization of BMMs towards the M1 phenotype is consistent with recent studies [25]. In addition, we found that TAB1-siRNA can suppress the polarization and inflammation caused by HG—levels of INOS, MCP-1 and IL-1β in macrophages under high glucose were inhibited by TAB1 siRNA. CD68 is used as a valuable cytochemical marker for immunostaining monocytes/macrophages in histochemical analysis of inflammation of tumor tissues [27]. Our in vivo results showed that CD68 and iNOS were overexpressed in the kidney after STZ treatment of mice. Inhibition of TAB1 by lentivirus lead to a reduction in infiltration and activation of M1 macrophages. In addition, TAB1 lentivirus markedly attenuated the upregulation of IL-1β and TNF-α induced by STZ. Additionally, the results of our study indicated that TAB1 lentivirus noticeably prevented the progression of DN—TAB1 lentivirus reduced the albuminuria excretion, tubulointerstitial damage index and glomerular mesangial expansion index in diabetic mice. These results indicate that TAB1 regulates the occurrence of macrophage polarization and inflammation, improving DN. However, the mechanisms behind this remain unclear.

It has been reported that many cancers have an altered metabolism, called the Warburg effect. These reports propose a neoteric form of metabolism. Cancer cells preferentially metabolize glucose through glycolysis, thereby producing lactic acid as an end product without using oxygen [28]. Under this transformation, tumor cells survive with optimized utilization of glucose. Increasing evidence suggests that the Warburg effect occurs in other situations. Activated inflammatory immune cells also exhibit metabolic features similar to glycoclastic cancer cells [29, 30]. M1 macrophage, which have a high energy demand, obtain energy from glycolysis to apidly provide ATP to promote inflammation. So macrophages activated by pro-inflammatory factors switch towards glycolysis, ignoring oxygen. For example, as a classical activator of M1 phenotype macrophages, lipopolysaccharide (LPS) has been reported to be involved in metabolic reprogramming of macrophages through increasing translation of HIF-1α mRNA, increasing expression of u-phosphofructokinase2 and inhibiting expression of Adenosine 5‘-monophosphate (AMP)-activated protein kinase, which contribute to macrophage glycolysis. Conversely, M2 phenotype macrophages appear to be more inclined towards oxidative phosphorylation—numerous processes that activate the glycolytic process in pro-inflammatory macrophages are decreased in M2 macrophages, which increasingly flux through OXPHOS [31, 32]. Interestingly, imbalance in glycolytic metabolites, such as succinate and lactate, can also interferes with inflammatory phenotypes via activating signalling pathways, post-translational mechanisms, epigenetic landscape and pos-transcriptionally regulate [33]. As the rate-limiting glycolytic enzymes, PFKFB3, HK1 and LDHA play a critical role in glycolysis and are over-expressed in glycoclastic cells [34–36]. Recent studies report that M1 macrophages express high levels of glycolytic enzymes and that HK1 regulates NLRP3 inflammasome activation, which subsequently regulates the production of IL-1β. PFKFB3 participates in the anti-viral response of macrophages. Current research also suggests that LDHA may be a key metabolic enzyme involved in regulation of inflammation [37]. Glycolysis promote the inflammation through multiple mechanism, metabolic reprogramming of macrophages promises to be a new target for macrophage-mediated inflammatory diseases. In our study, lactic acid production and glucose absorption test results showed that lactate production and glucose uptake in the HG group were significantly higher when compared with the control group. Compared with the HG group, lactic acid production and glucose absorption capacity of the TAB1 siRNA + HG group were decreased, and this difference was statistically significant. In the HG group, we also observed stronger staining for HK1, PFKFB3 and LDHA when compared with controls, and HG-induced BMMs treated with TAB1 siRNA showed a reduction in HK1, PFKFB3 and LDHA staining. Results of Western blots and RT-PCR also showed the same trend. The protein levels and mRNA of glycolytic enzymes HK1, PFKFB3 and LDHA in HG-induced BMMs were distinctly higher than the levels in the control group. In the TAB1 siRNA + HG group, they were significantly weaker than those in the HG group, which proved that the glycolysis of HG-induced M1 macrophages was enhanced and expression of glycolytic enzymes HK1, PFKFB3 and LDHA were up-regulated in an HG environment. TAB1 silencing inhibited the enhancement of glycolysis under HG conditions. Similarly, in vivo, immunohistochemical and Western blotting demonstrated that STZ-induced diabetic mouse kidneys showed higher levels of HK1, PFKFB3 and LDHA, and these STZ-induced glycolytic enzymes were notably inhibited by TAB1 lentivirus. Focusing on macrophages in renal tissue, immunohistochemical double-staining revealed that TAB1 inhibition decreased STZ-induced glycolysis of renal macrophages. Therefore, Macrophages enhance glycolysis upon activation by glucose dysregulation, and silencing of TAB1 inhibits macrophage glycolysis while reducing inflammation in vivo and in vitro.

NF-κB is a recognized downstream signaling target molecule of TAB1/TAK1. After Toll-like receptor signal transduction, TAK1/TAB1 mediates NF-κB p65 nuclear entry through IKKα/β, inducing further inflammation [9]. Emerging evidence points to NF-κB control over the HIF signaling pathway and interaction between the HIF signaling pathway and the NF-κB signaling cascade [14]. It has been confirmed that NF-κB controls HIF-1α transcription in macrophages activated by LPS through ChIP [38]. High expression of specific transcription factors, principally HIF-1α, NF-κB and Octamer Binding Transcription Factor 1, contribute to the Warburg effect. In other words, HIF-1α is involved in regulating the energy metabolism of cells and makes cells more inclined to utilize glucose through glycolysis. Simultaneously, studies have shown that patients with DN have higher HIF-1α levels and sodium-dependent glucose transporters 2 inhibitors, recently used to treat DN, also target HIF-1α [12]. In the classical inflammatory cells, HIF-1α is involved in glycolysis and has a role in inflammation. But whether HIF-1α can be driven by high glucose via NF-κB has not been studied in depth. Therefore, it is worth exploring thoroughly whether TAB1 regulates macrophage glycolysis through TAB1/NF-κB/HIF-1α and mediate inflammation in M1 phenotype macrophage. In our study, we observed that the green fluorescence of NF-κB p65 in the control group was mainly located in the cytoplasmic region. In the HG group, the fluorescent of NF-κB p65 was mostly found in the nuclear region, and treatment with TAB1 siRNA significantly inhibited the presence of green fluorescence in the nucleus. Western blotting revealed that the levels of TAB1, NF-κB p65 and HIF-1α were increased significantly in the HG group. In the TAB1 siRNA + HG group, TAB1, NF-κB and HIF-1α protein expression both decreased, in contrast to the HG group. In vivo, the expression of TAB1, p-p65 and HIF-1α were measured in mouse kidneys. Western blot results showed that although the protein level of p65 remained unchanged, the expression of p-p65 was increased in STZ-induced diabetic mice, while TAB1 lentivirus decreased its expression significantly—this is consistent with the results of P65 nuclear expression in vitro. The same is true of the expression trends for TAB1 and HIF-1α. Results from ChIP showed specific binding of NF-κB to HIF-1α in HG-induced macrophages, confirming the potency of TAB1/NF-κB regulating HIF-1α. The above results suggest that TAB1 is involved in the regulation of NF-KB and HIF-1α to a certain extent, and this may be closely related to glycolysis and activation of macrophages.

At present, new developments in the treatment of DN include treatment options such as MCP-1 inhibitors that inhibit macrophages and inflammation, but more precise treatment targets are still urgently needed. Our study demonstrates that glucose dysregulation can initiate macrophage glycolysis and M1 polarization, causing the release of inflammatory factors. These effects are closely related to aggravation of DN. Downregulation of TAB1 signaling reverses glycolysis and M1 polarization of macrophages, reducing inflammation, improving the clinical and pathological manifestations of DN. In summary, our study proposes that the TAB1/NF-κB/HIF-1α signaling pathway mediates the glycolysis and polarization of macrophages to promote inflammation in DN.

## Electronic supplementary material

Below is the link to the electronic supplementary material.

Supplementary file1 (DOC 9750 kb)
