# Supplementary material for: TAB1 regulates glycolysis and activation of macrophages in diabetic nephropathy
Source: Inflamm Res. 2020 Oct 12;69(12):1215–34. doi: 10.1007/s00011-020-01411-4 (PMC7658079; doi:10.1007/s00011-020-01411-4)
Supplement: Supplementary file 1 — Supplementary file1 (DOC 9750 kb) [file 11_2020_1411_MOESM1_ESM.doc]

**supplementary materials**

F4/80 HK1 DAPI Merge


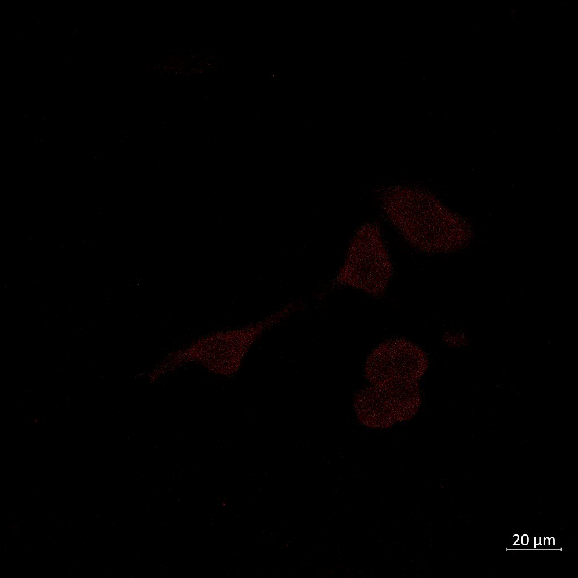

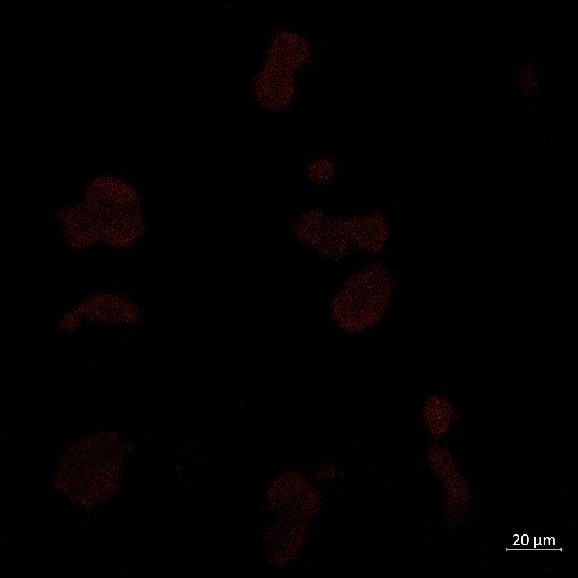

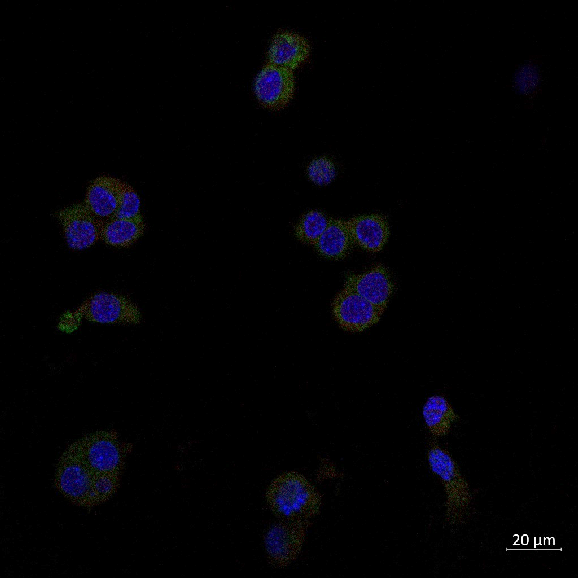

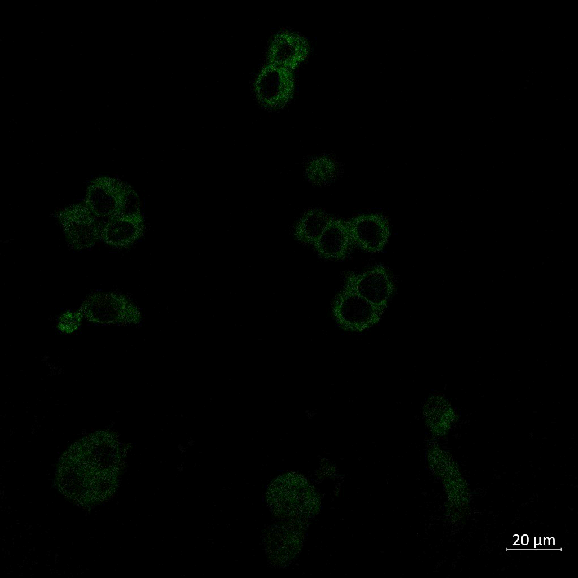

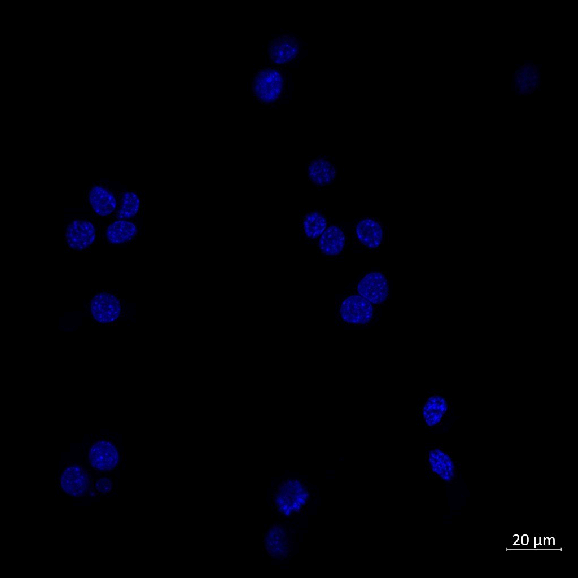

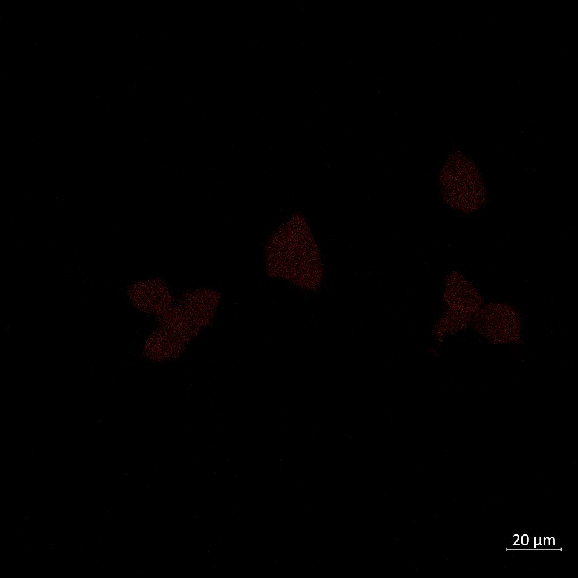

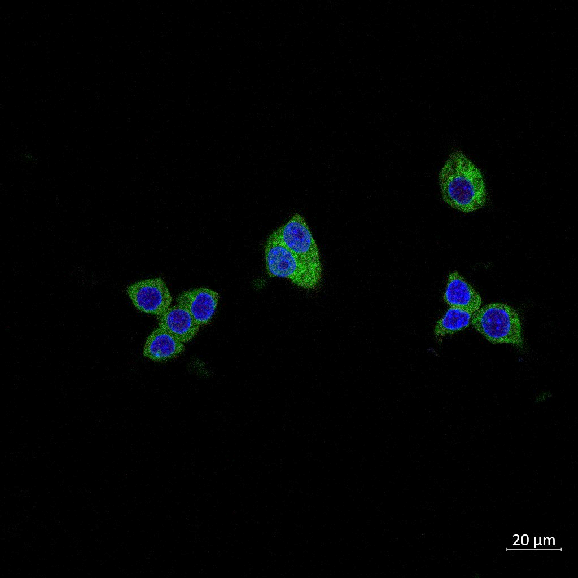

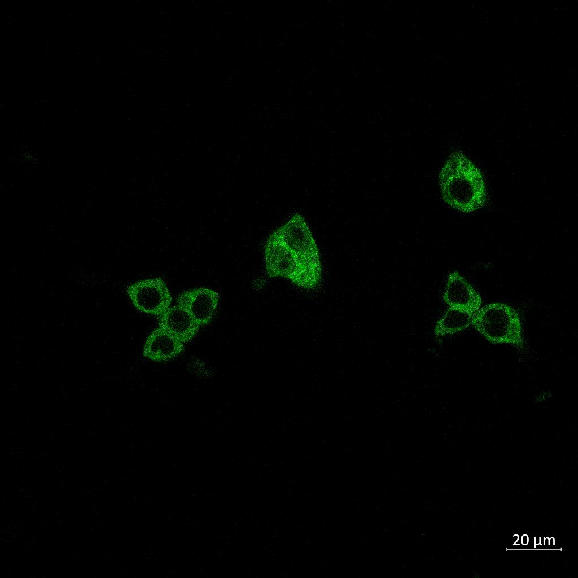

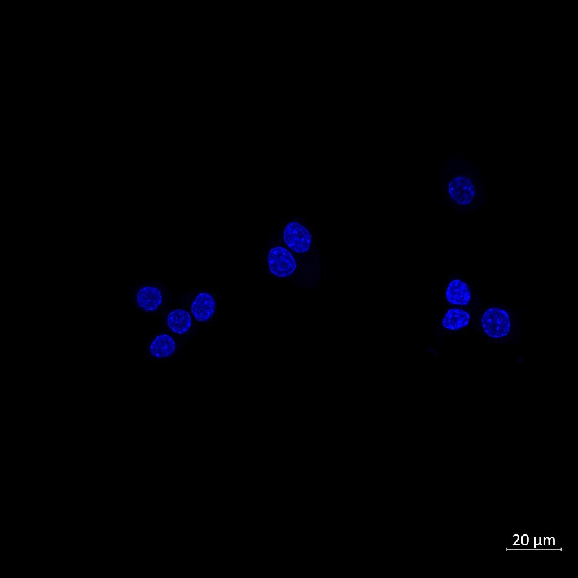

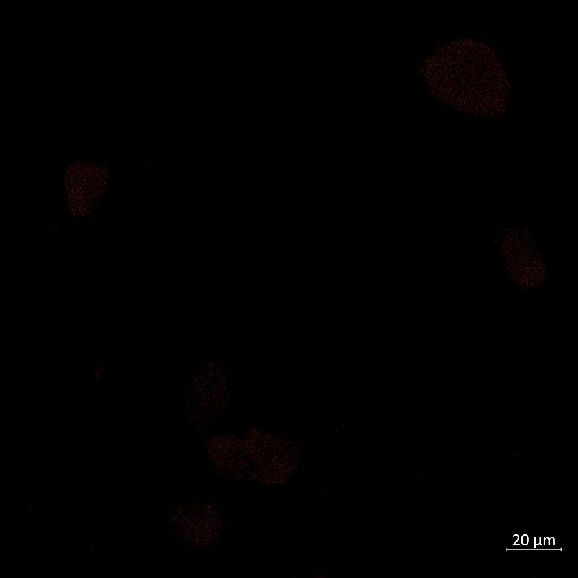

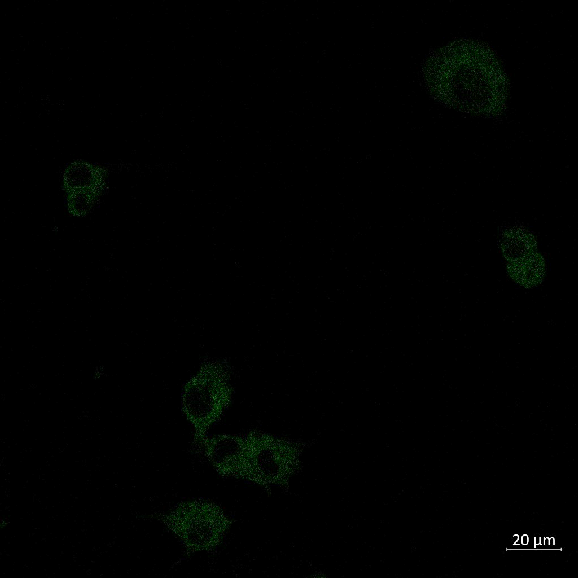

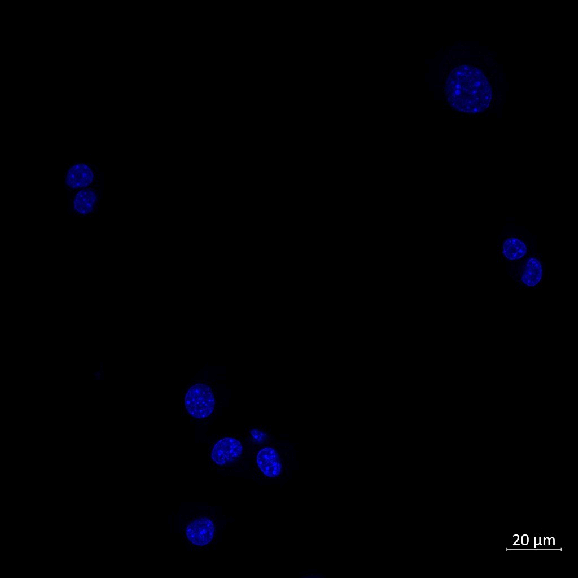

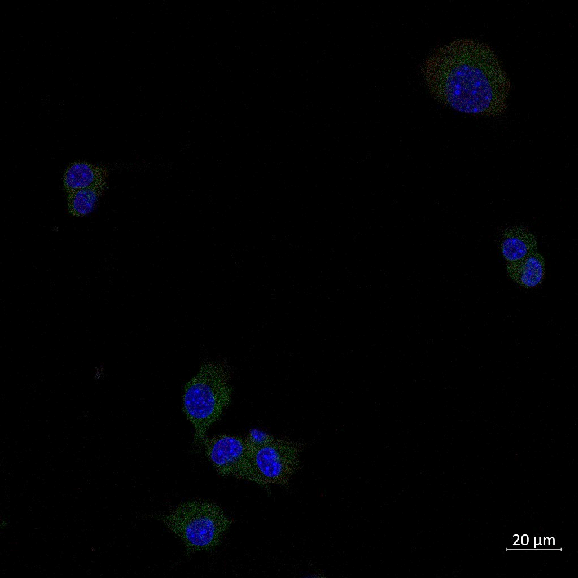

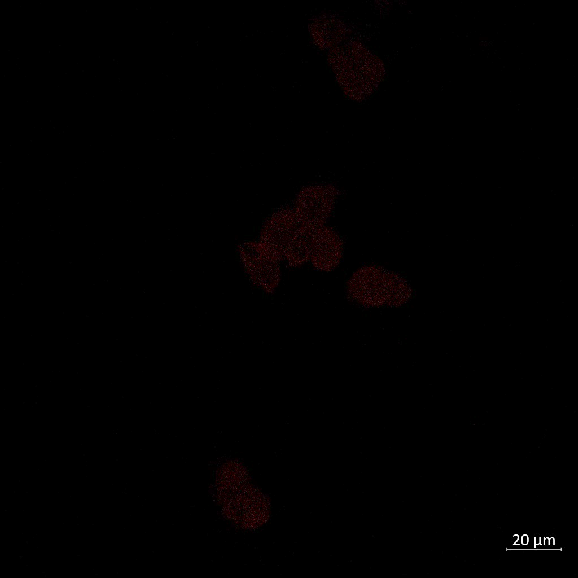

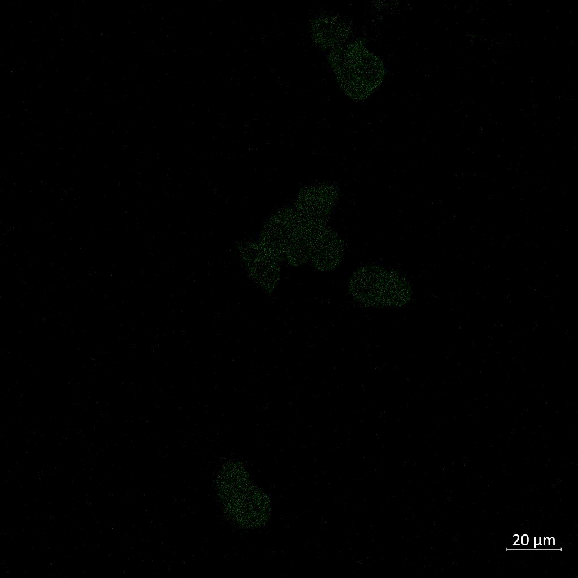

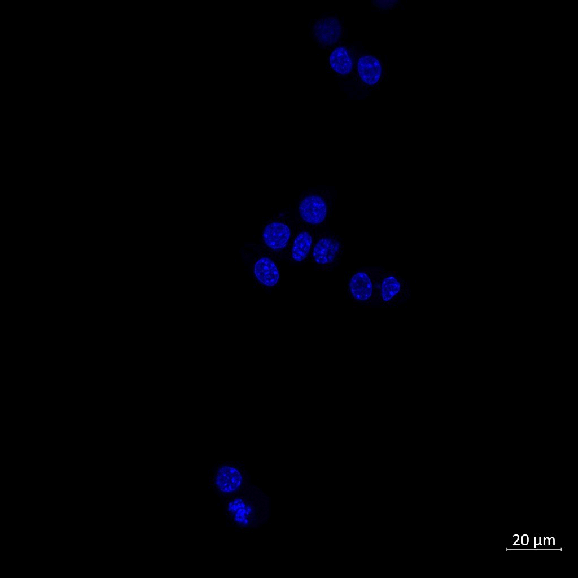

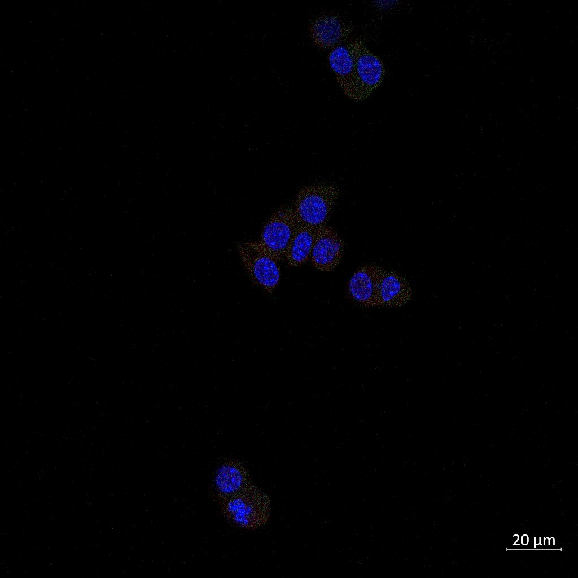

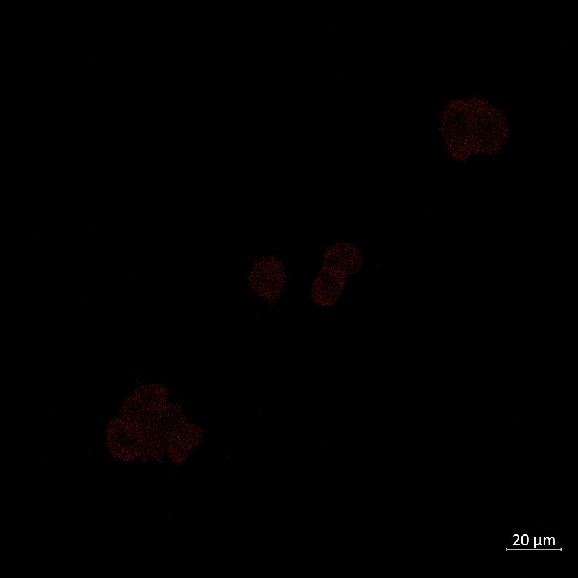

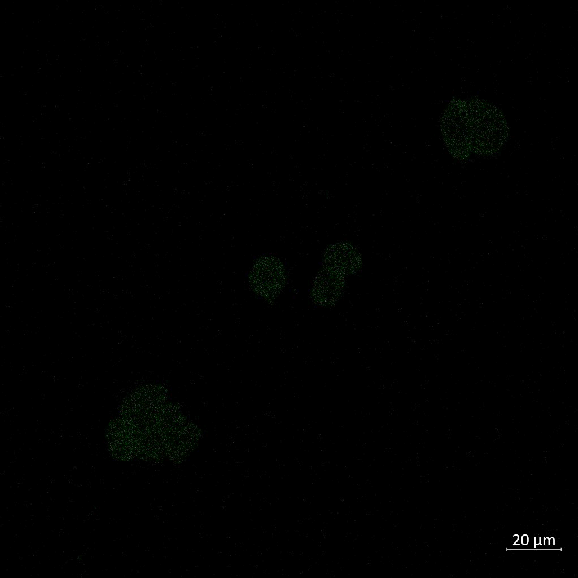

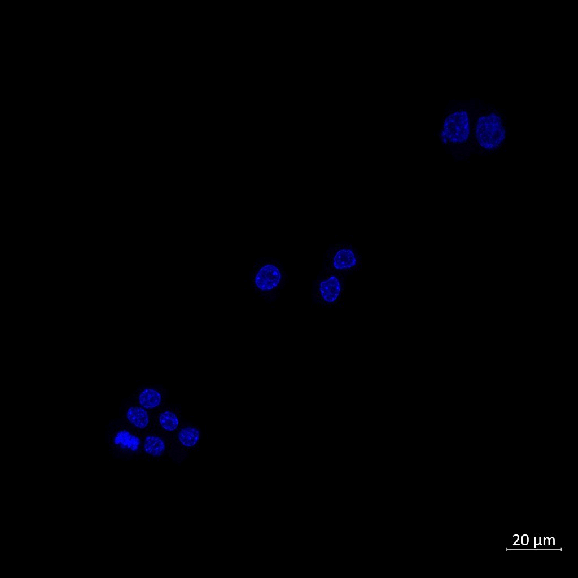

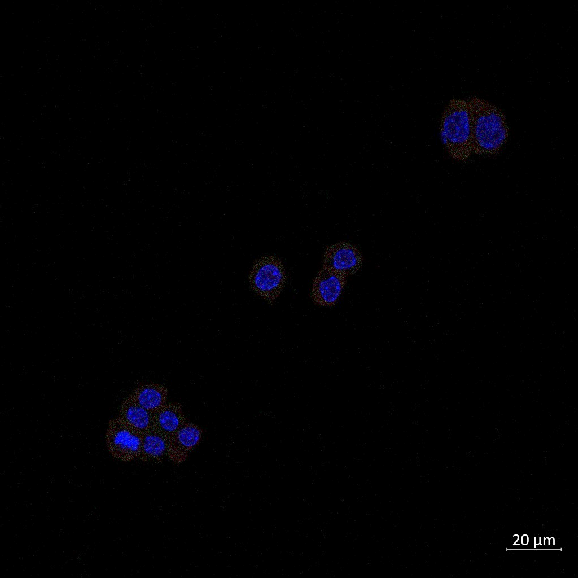

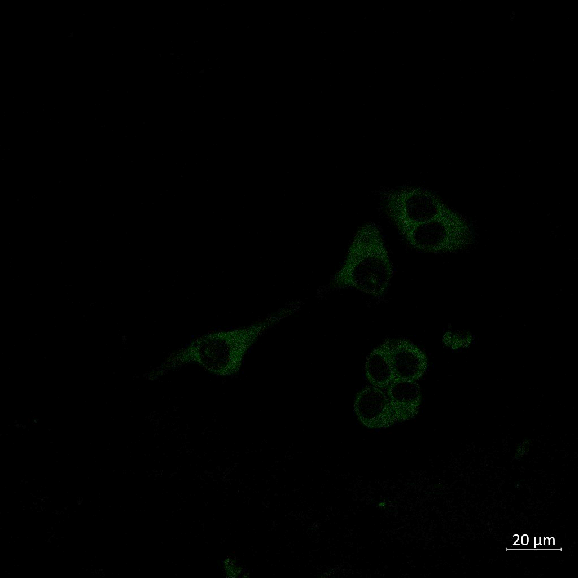

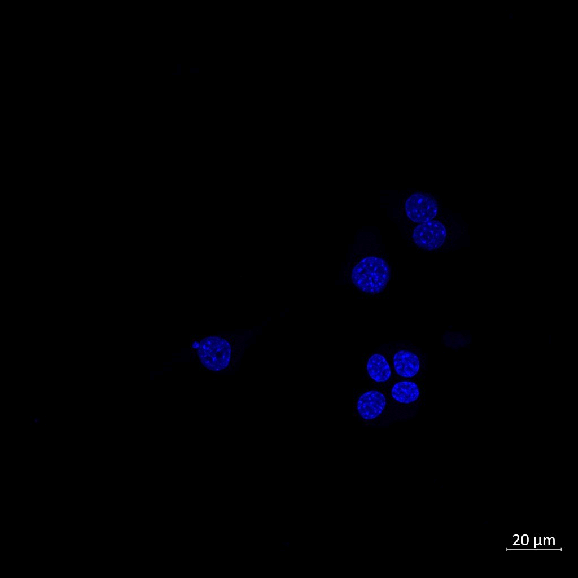

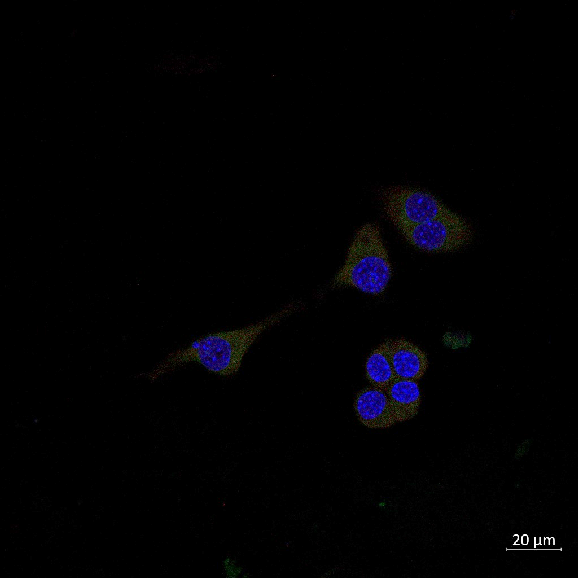


D-Mannitol

control siRNA

control

HG

TAB1 siRNA

HG+TAB1 siRNA


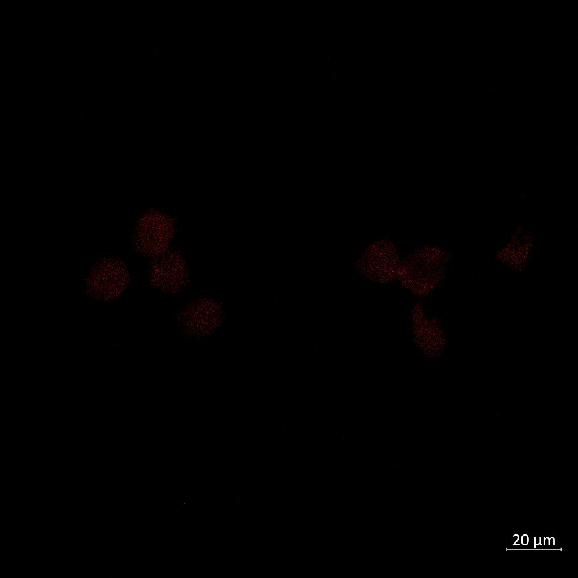

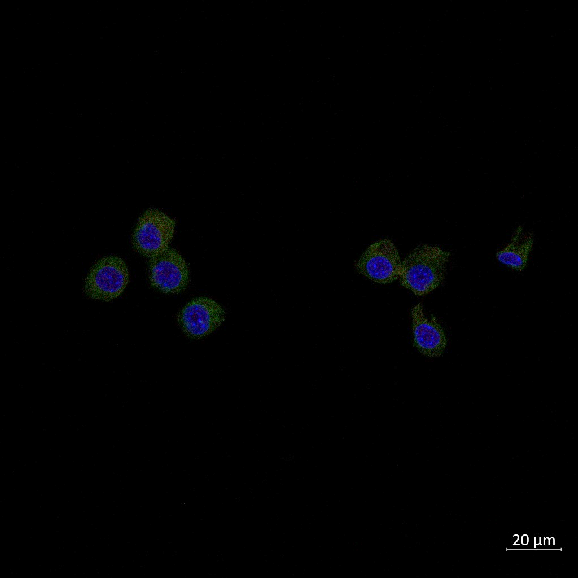

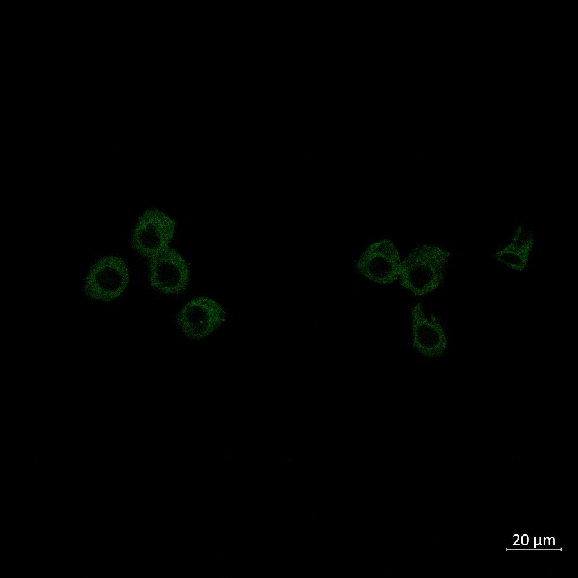

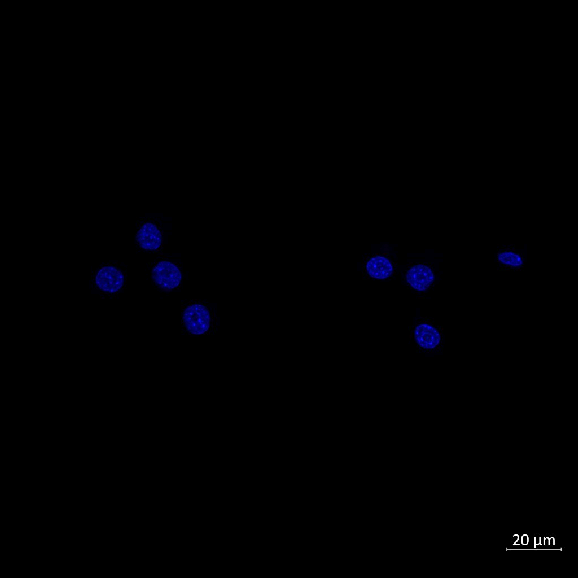

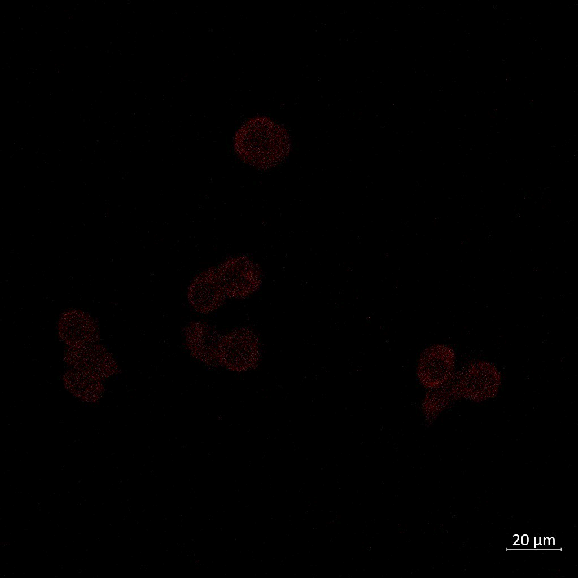

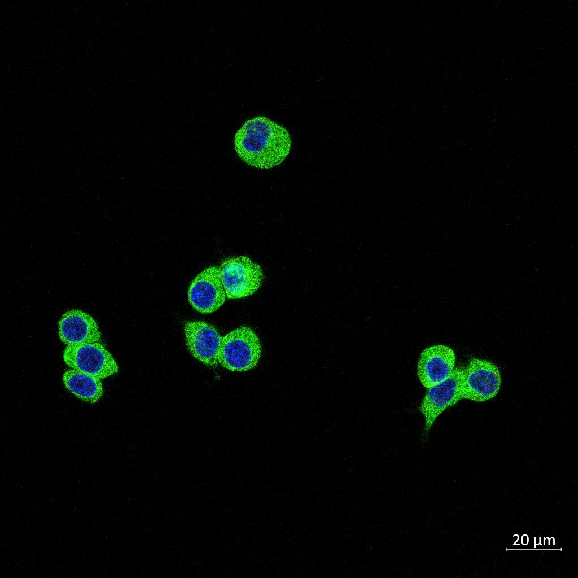

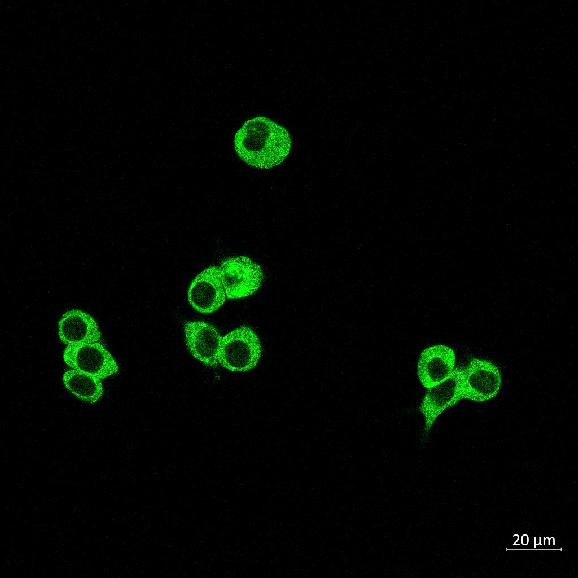

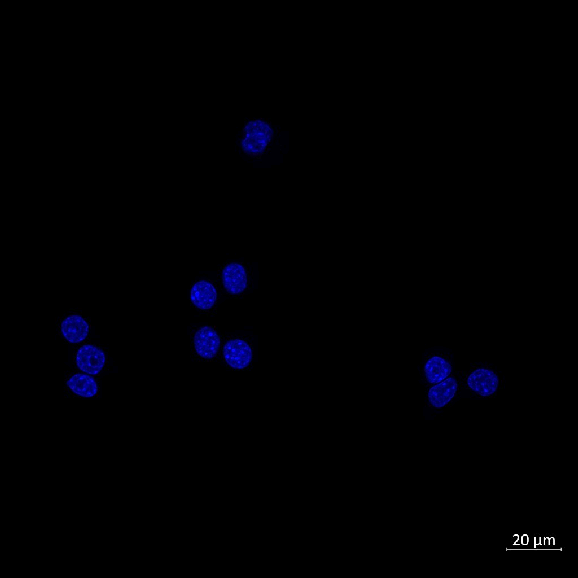

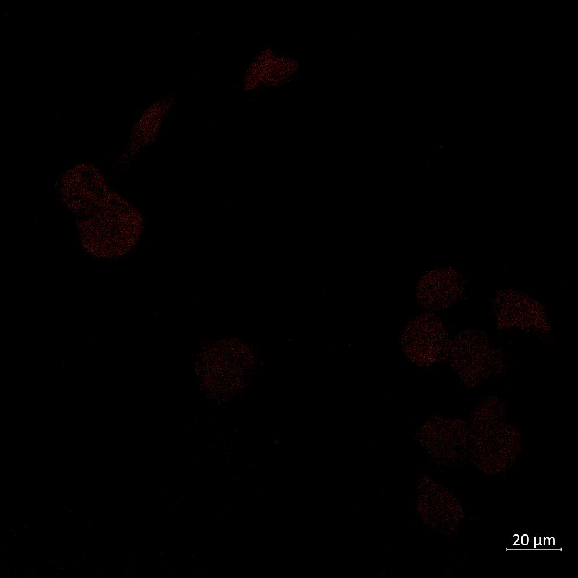

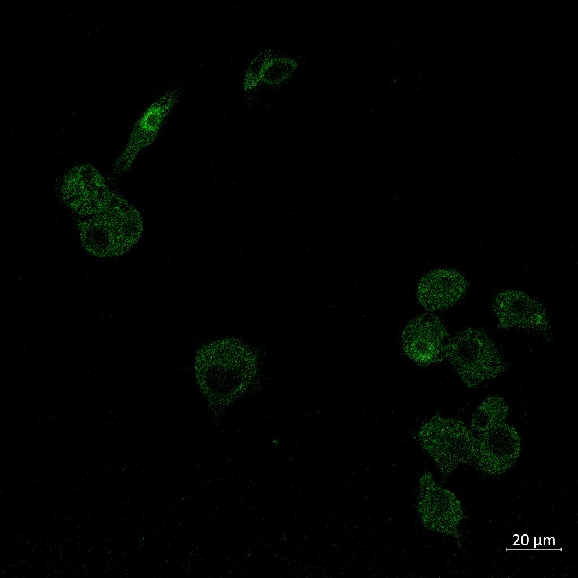

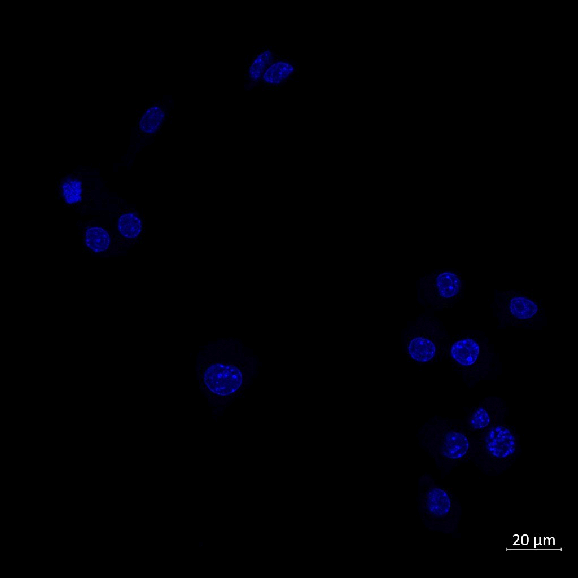

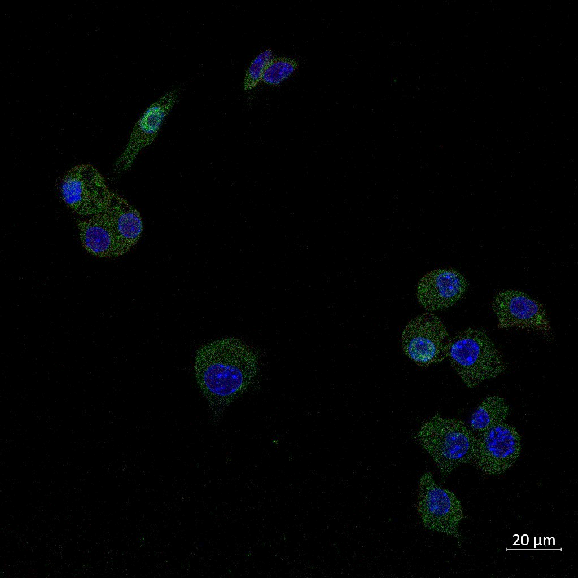

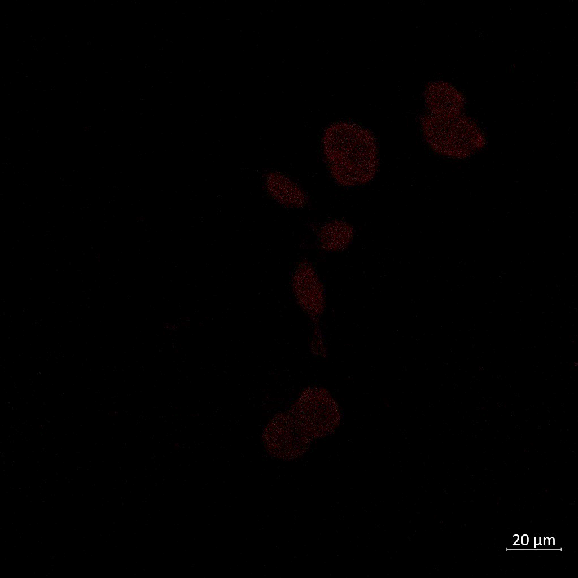

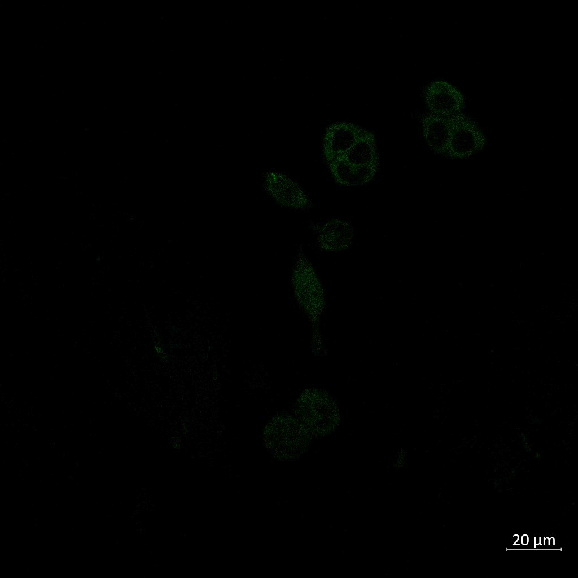

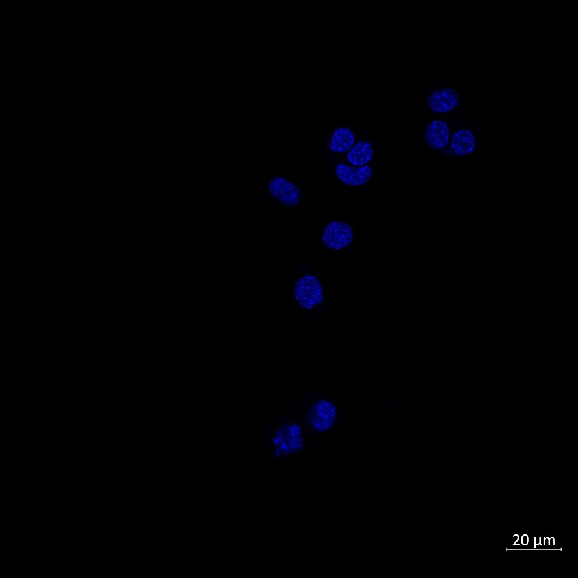

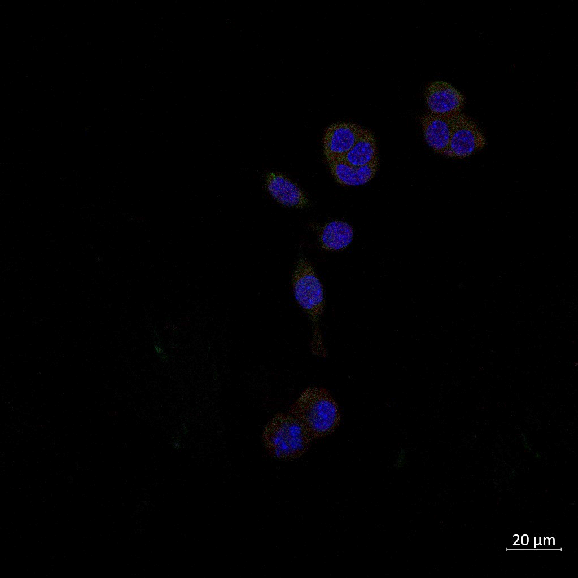

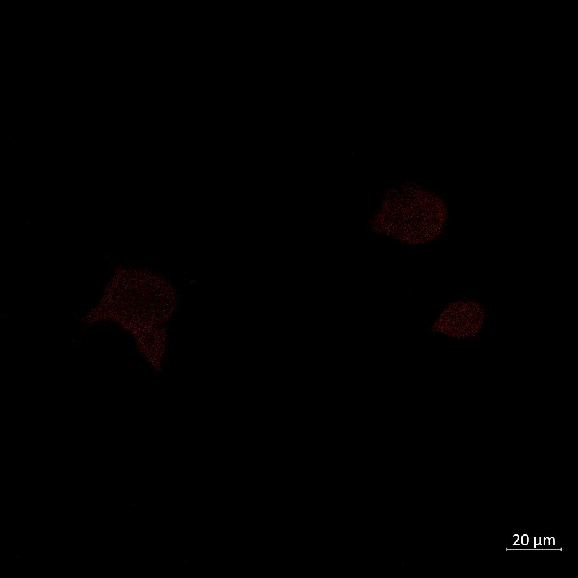

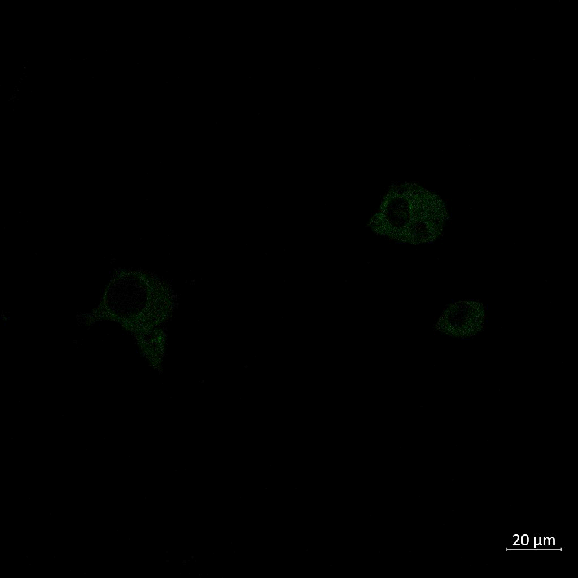

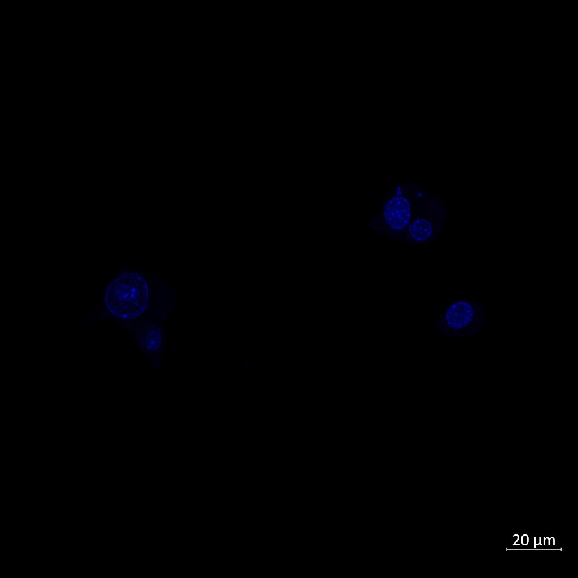

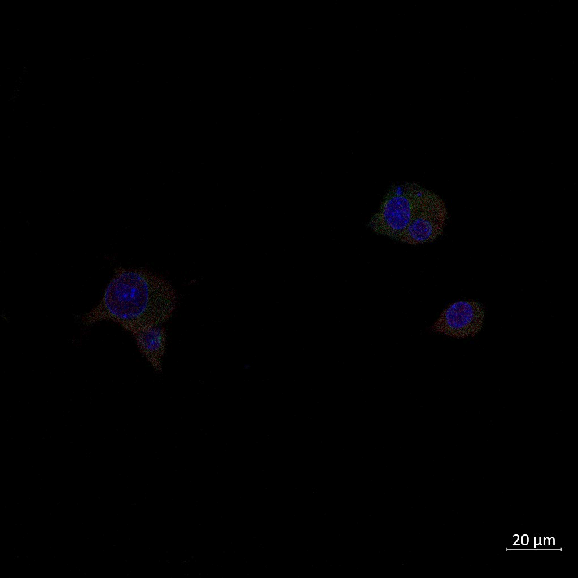

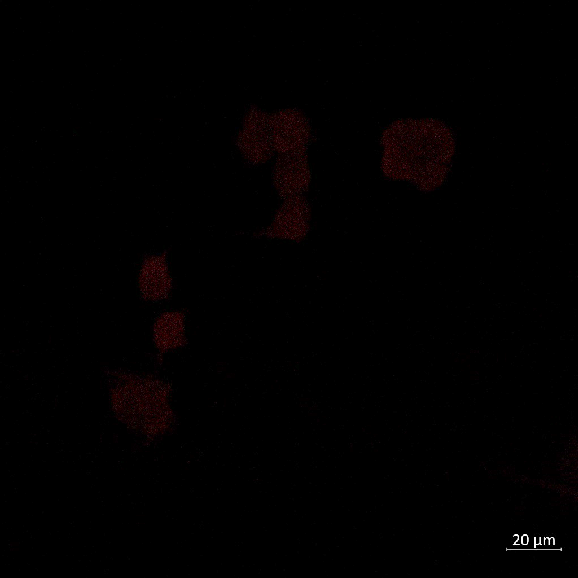

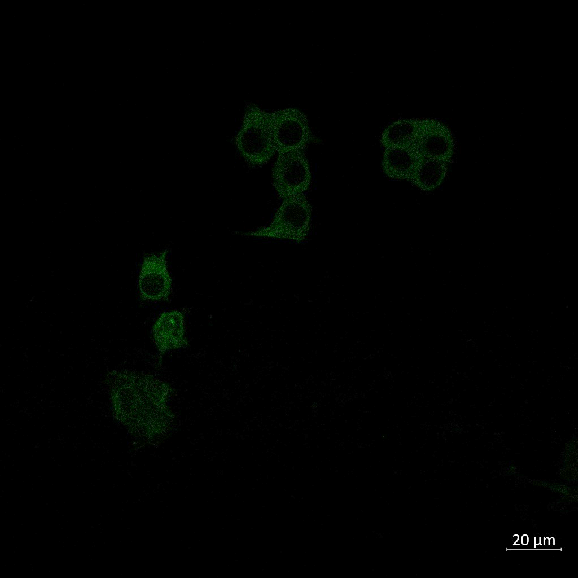

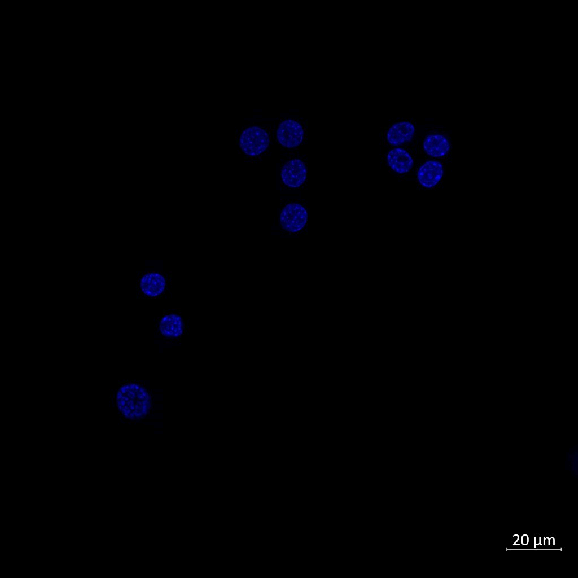

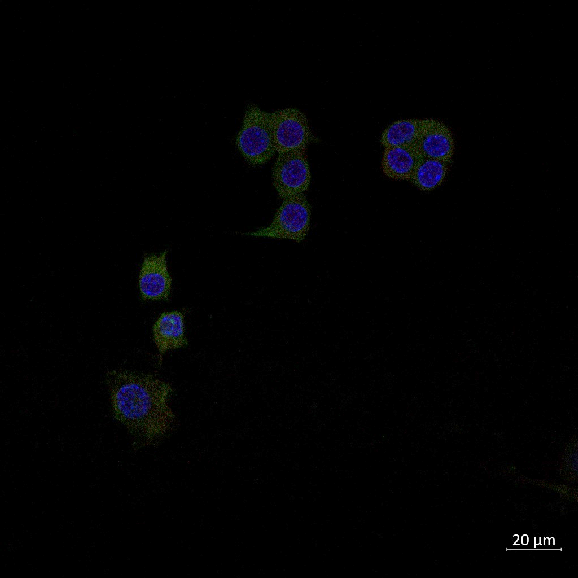


D-Mannitol

control siRNA

control

HG

TAB1 siRNA

HG+TAB1 siRNA

F4/80 PFKFB3 DAPI Merge


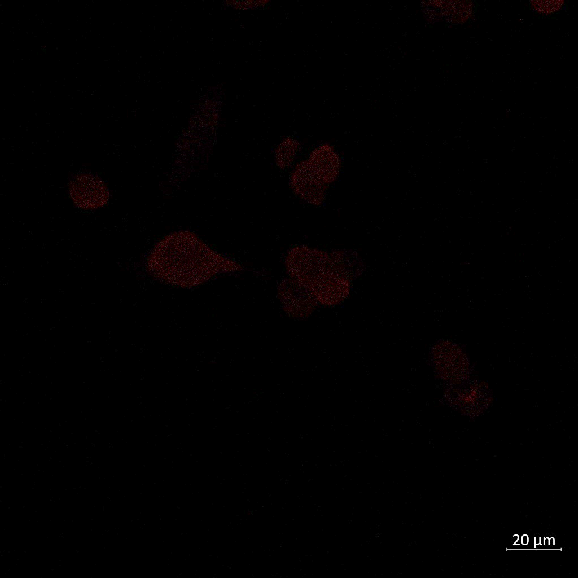

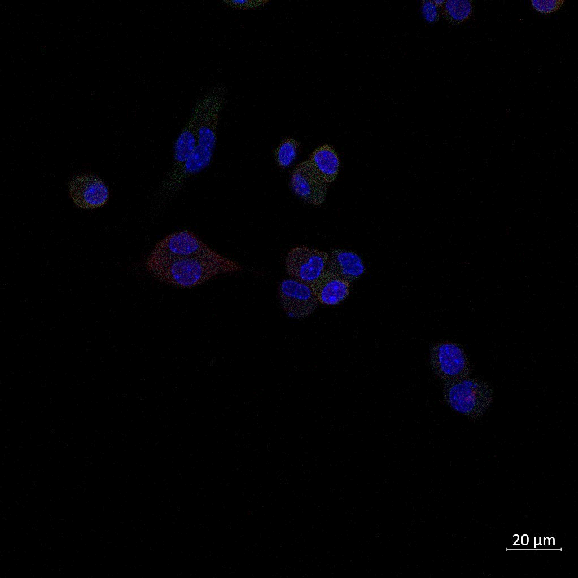

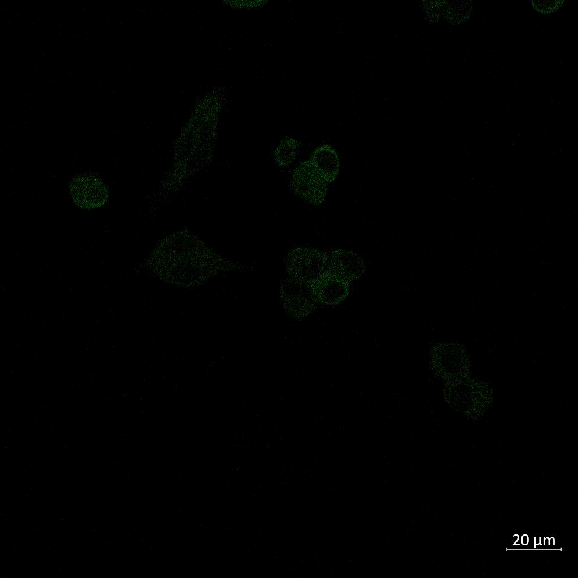

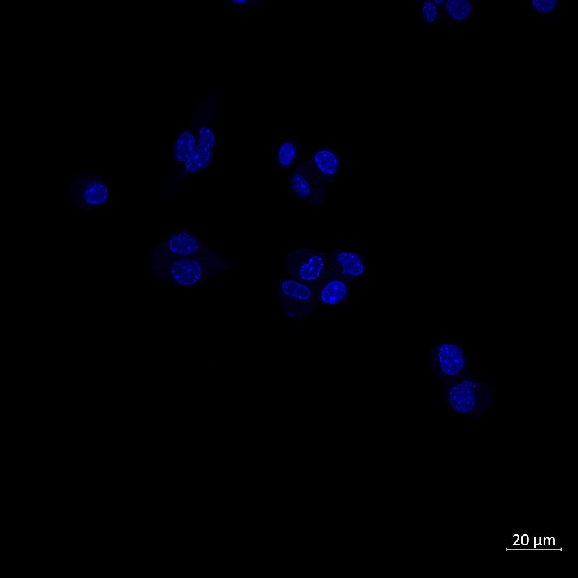

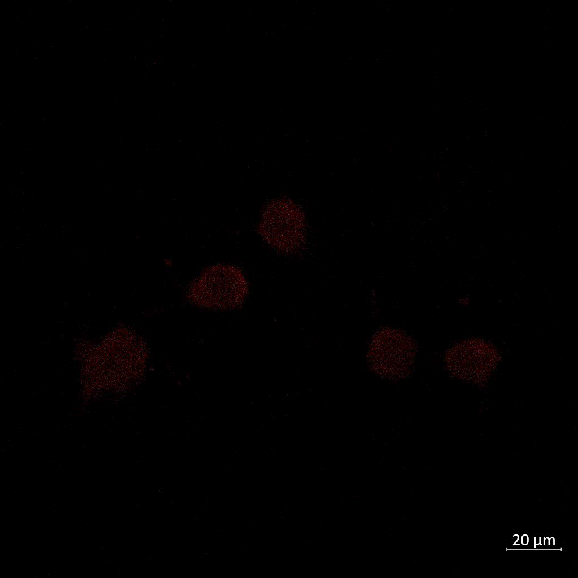

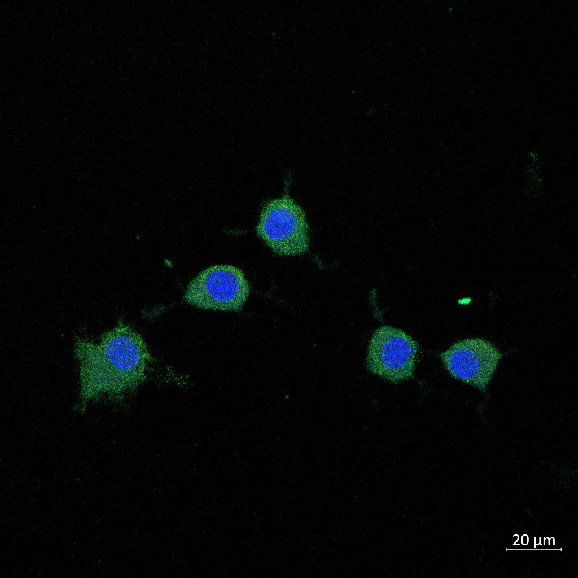

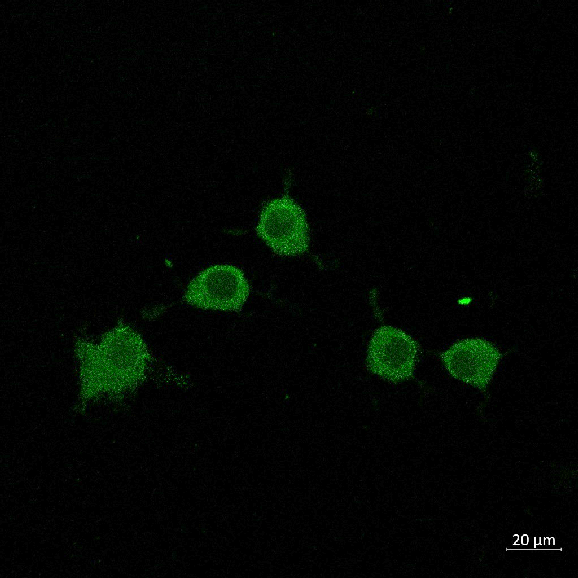

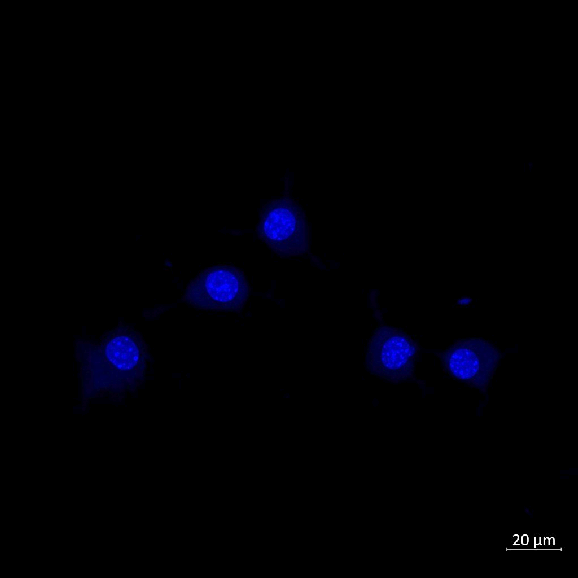

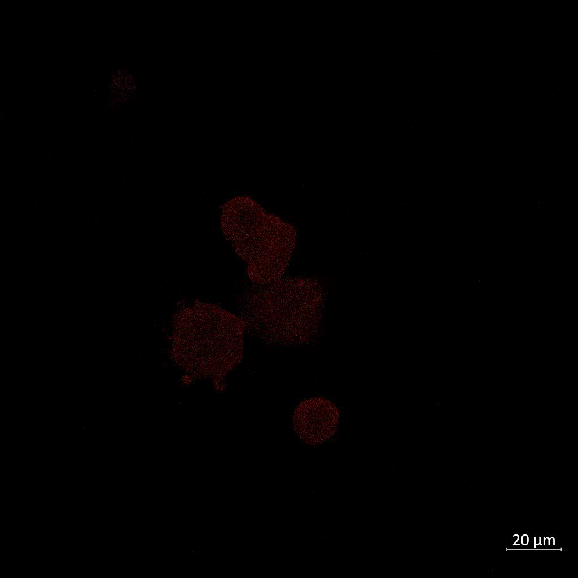

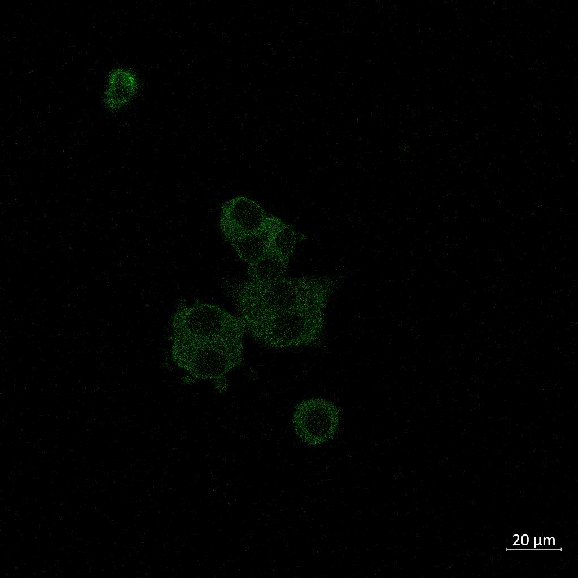

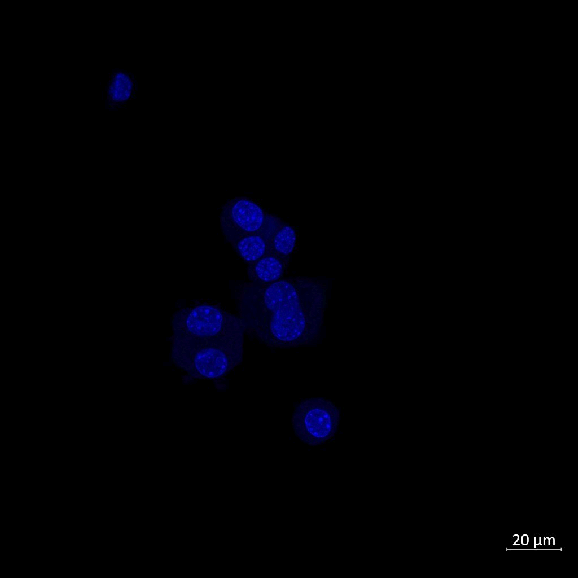

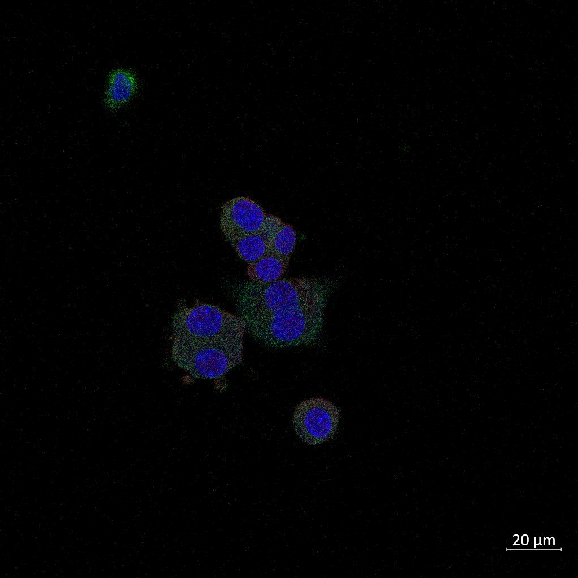

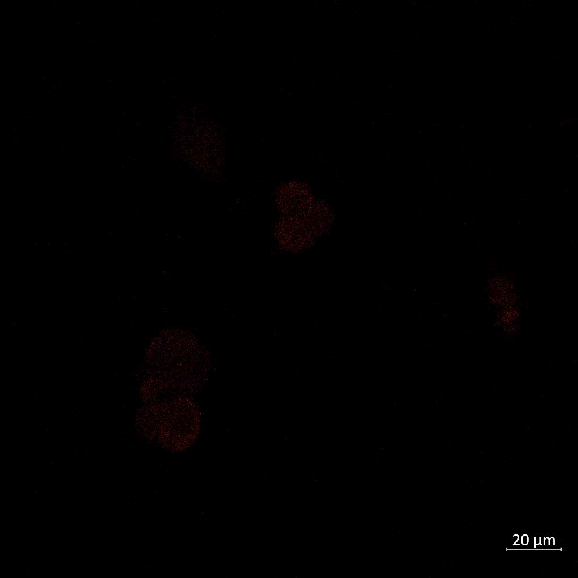

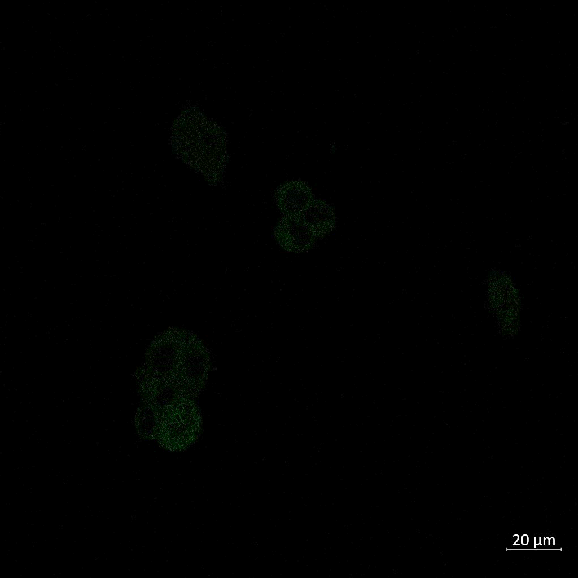

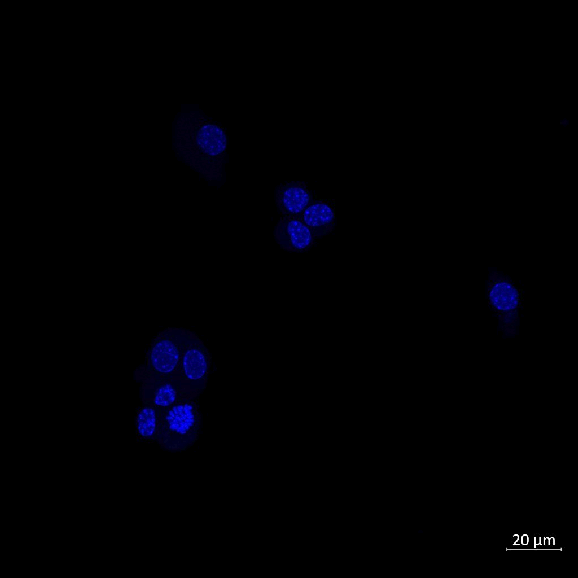

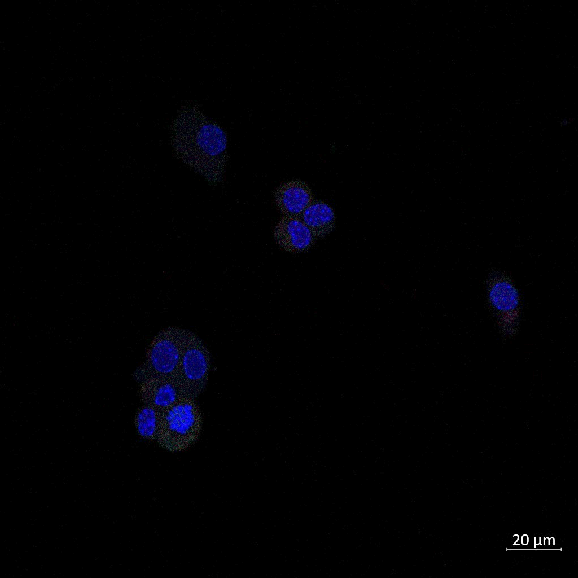

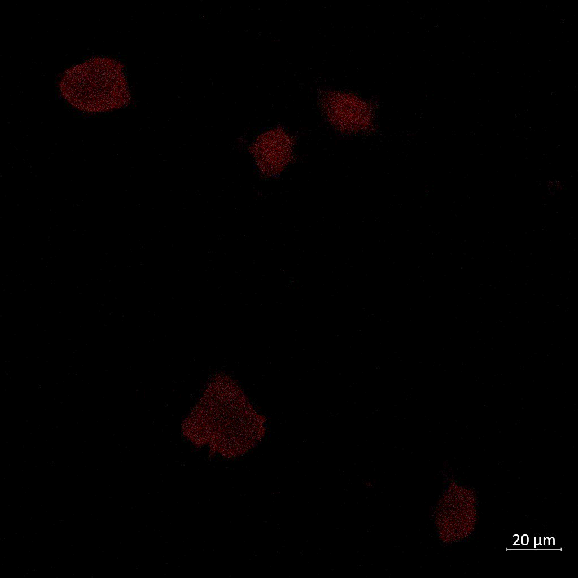

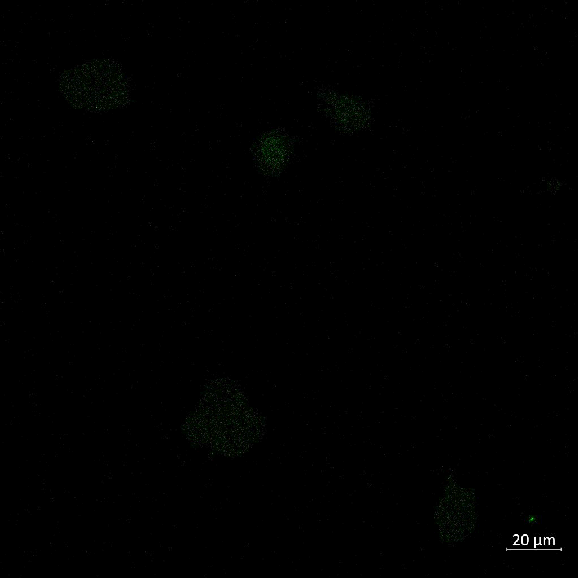

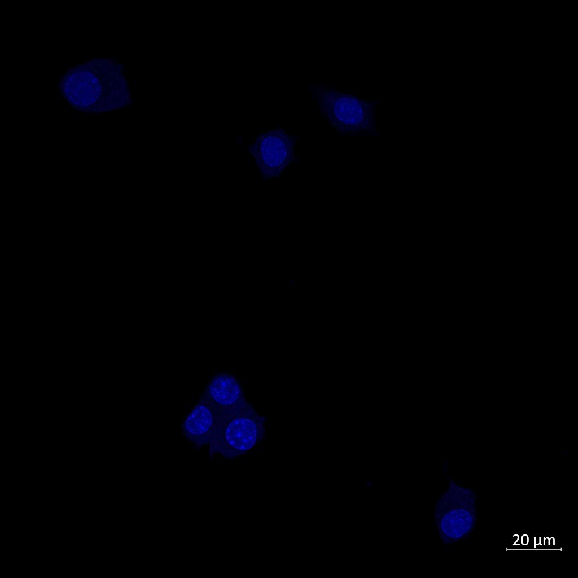

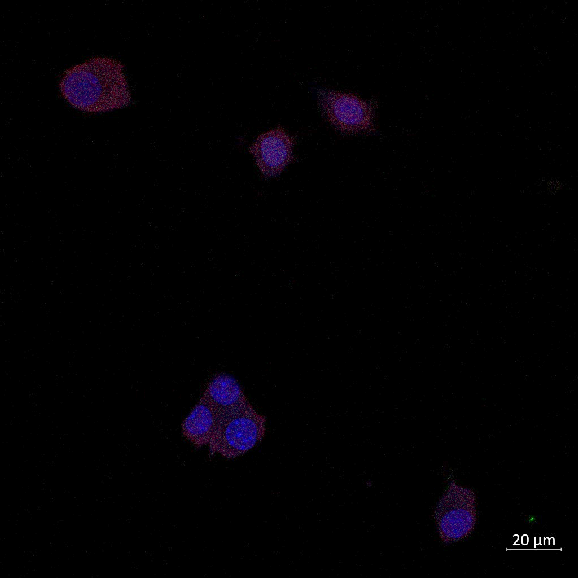

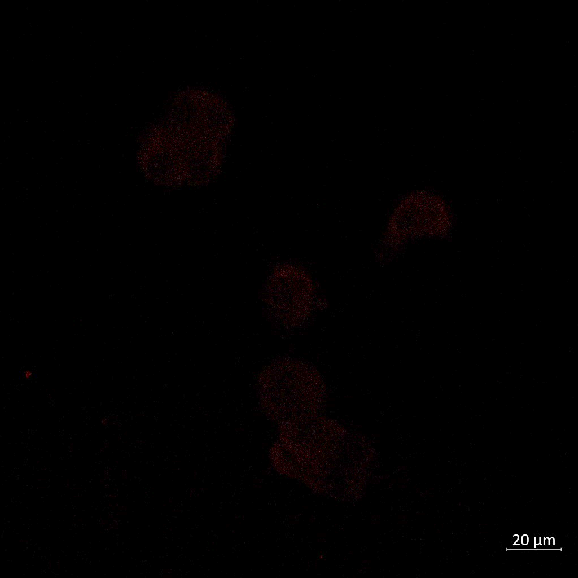

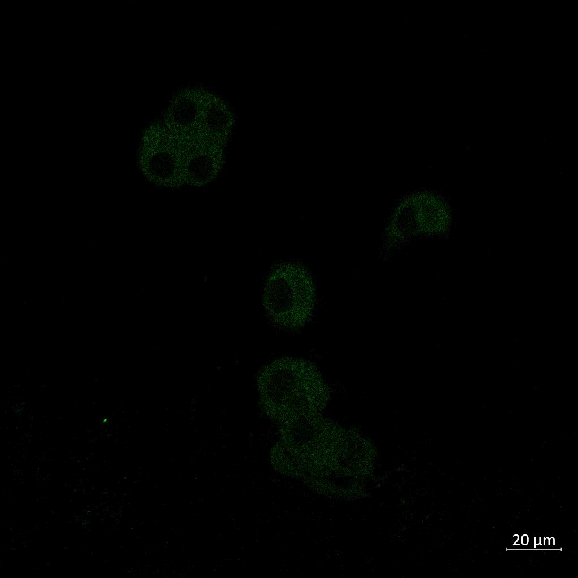

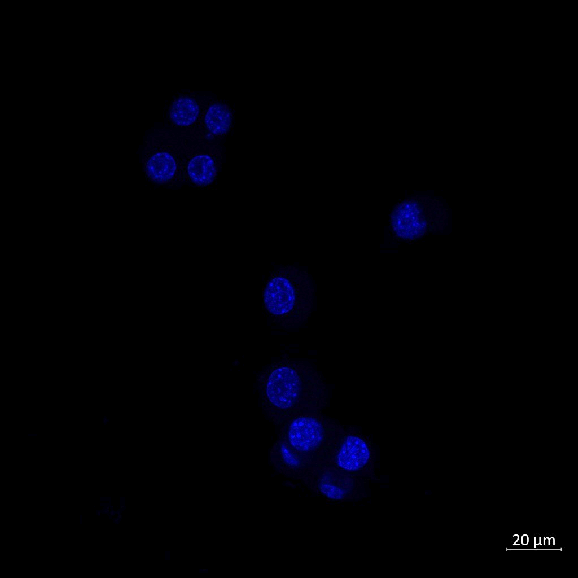

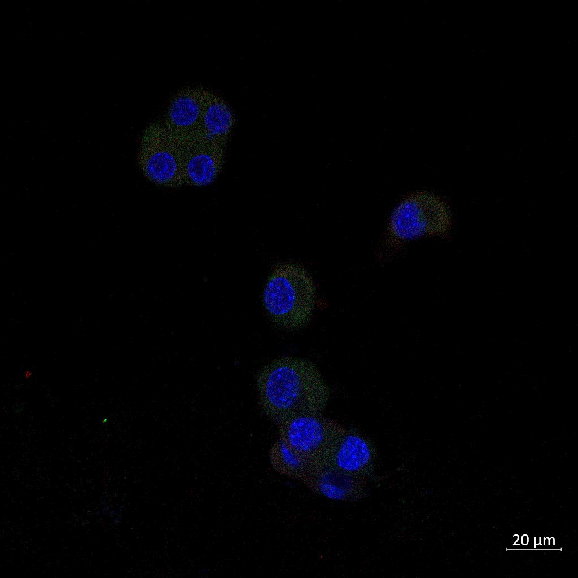


D-Mannitol

control siRNA

control

HG

TAB1 siRNA

HG+TAB1 siRNA

F4/80 LDHA DAPI Merge


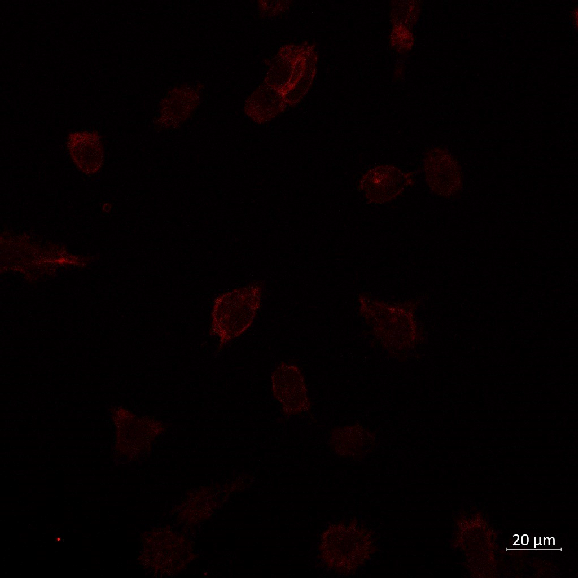

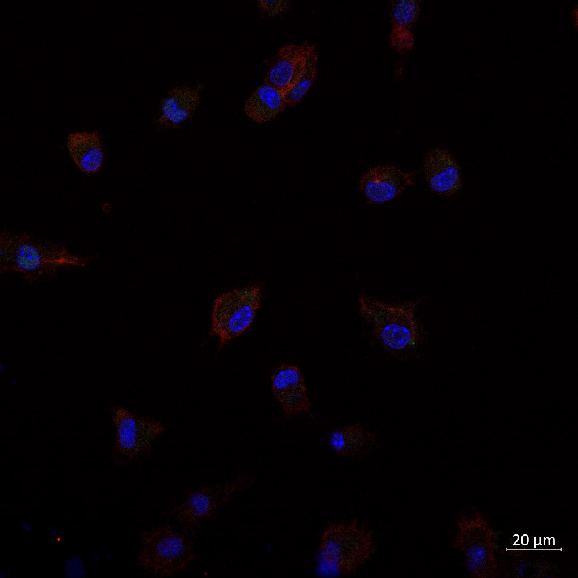

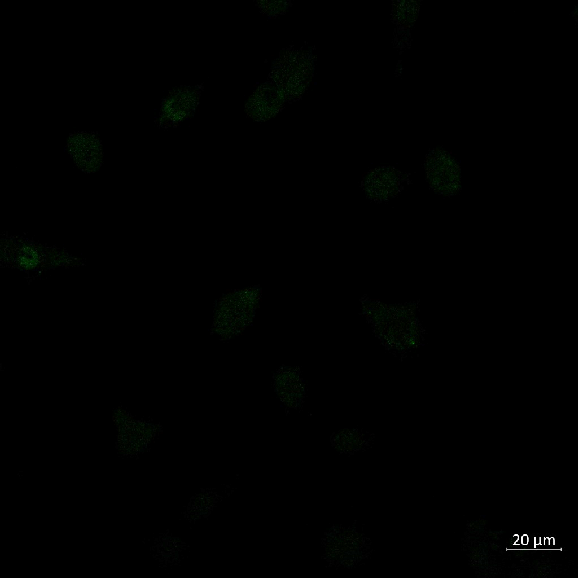

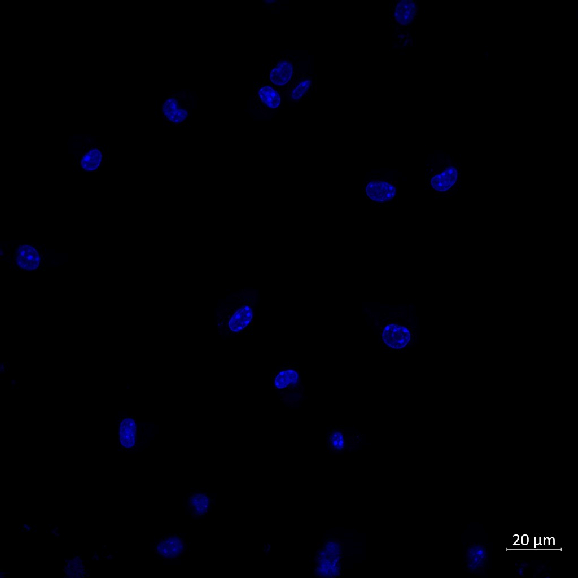

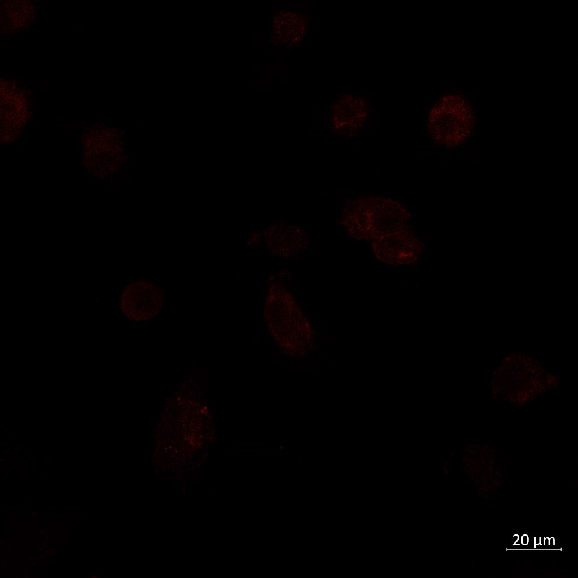

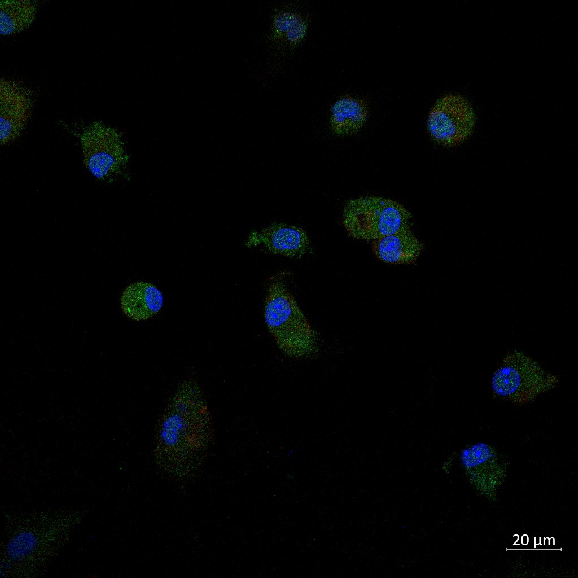

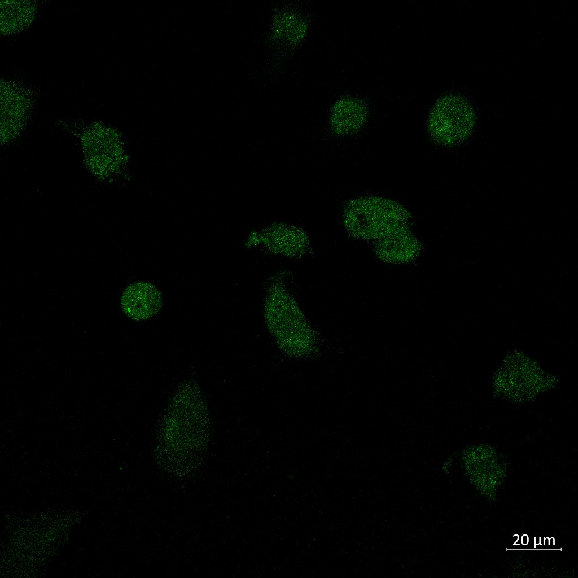

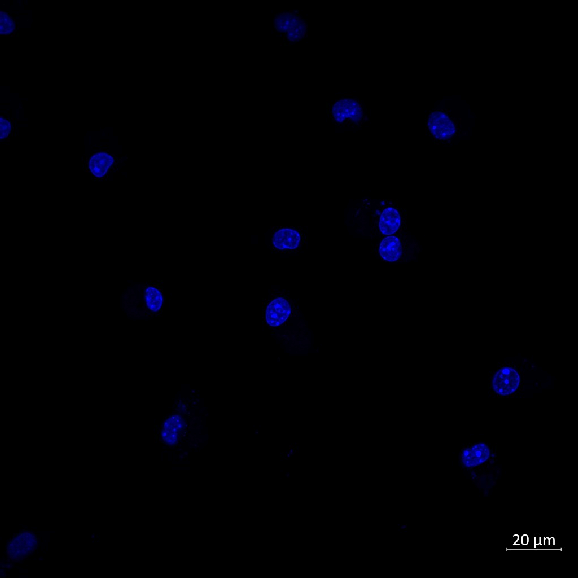

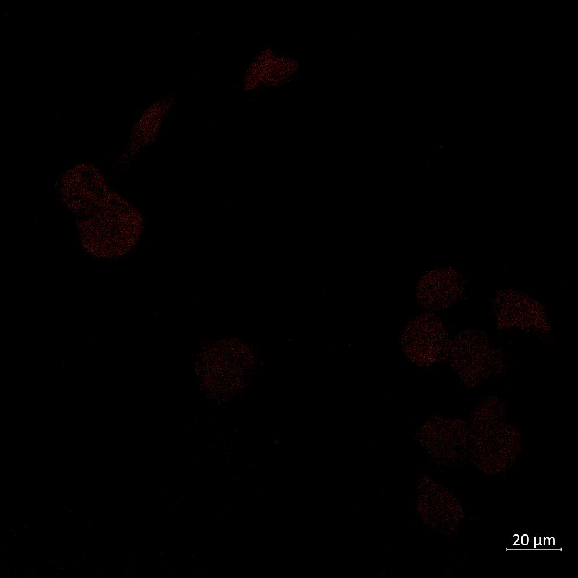

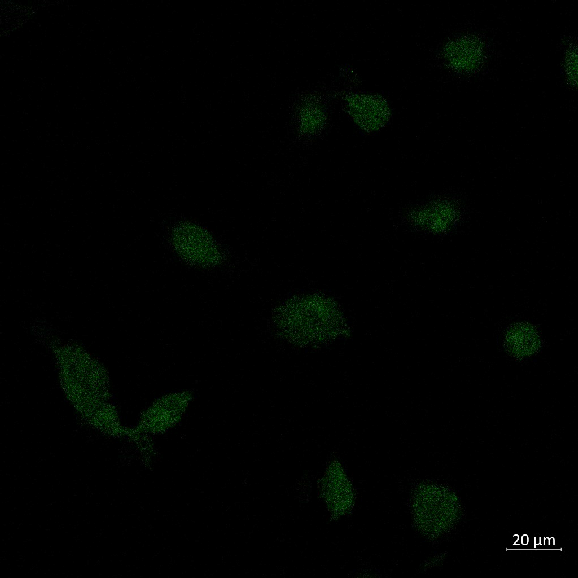

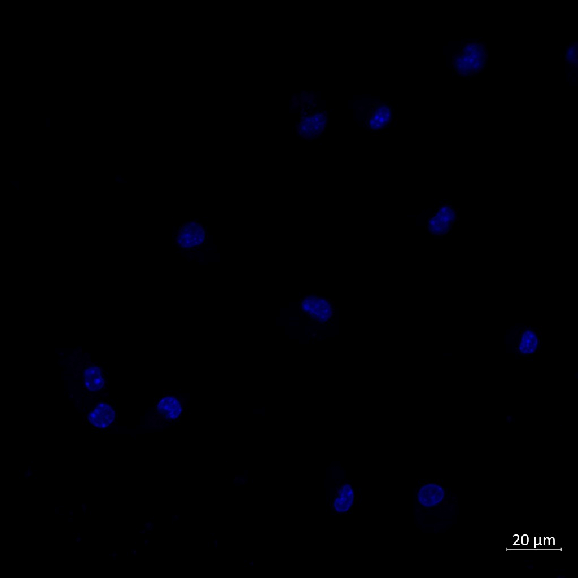

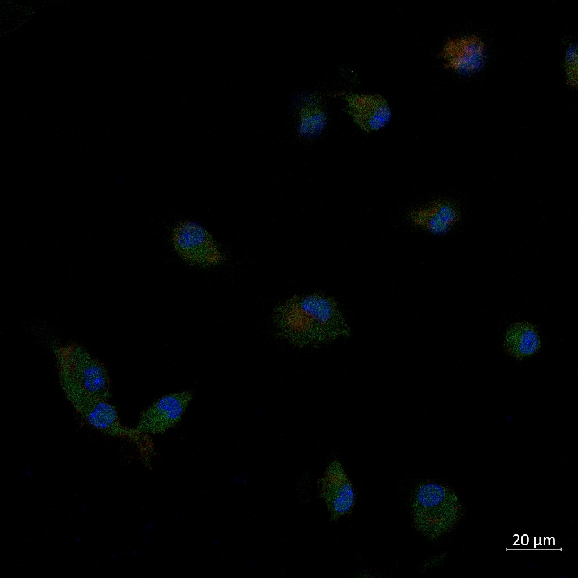

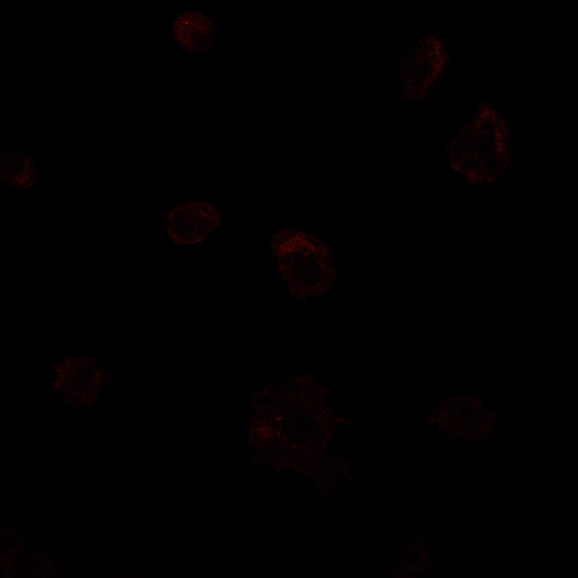

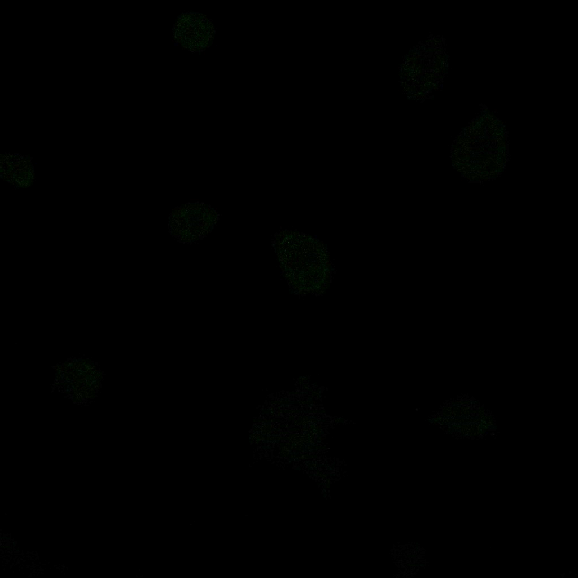

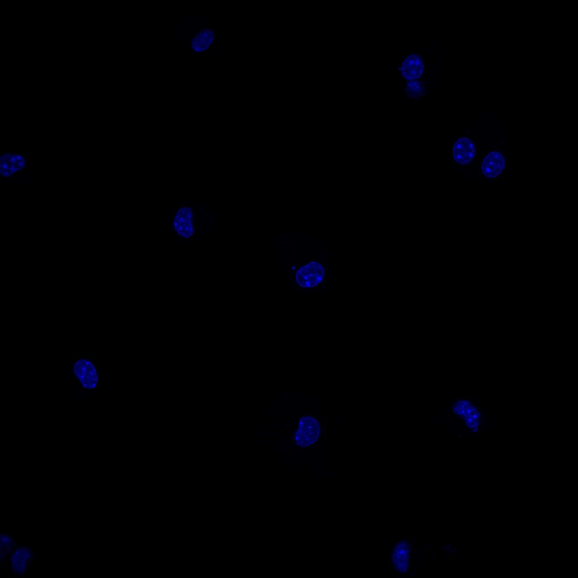

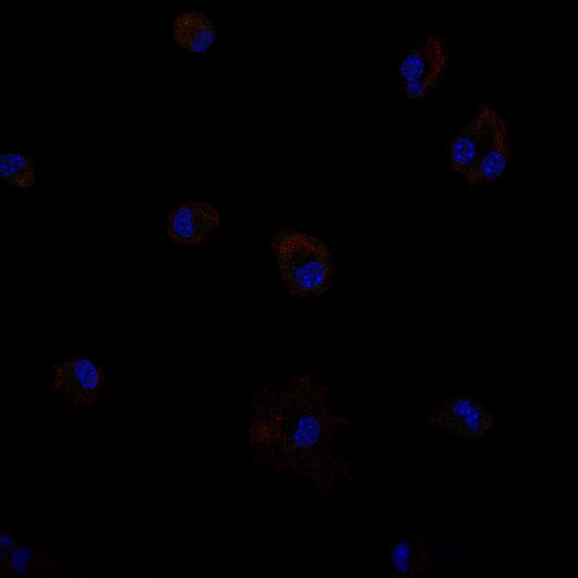

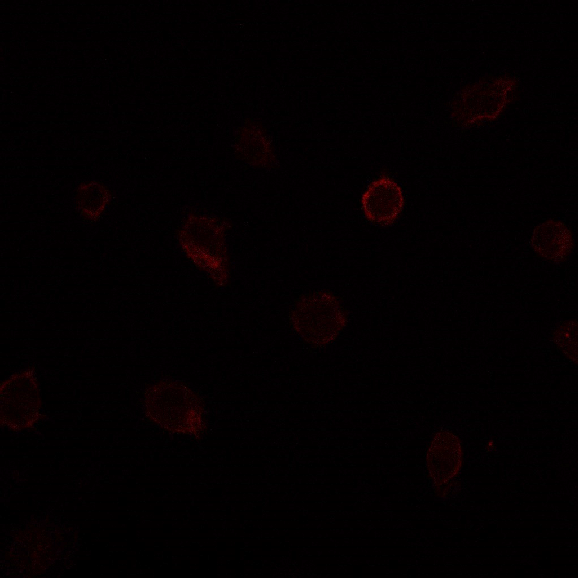

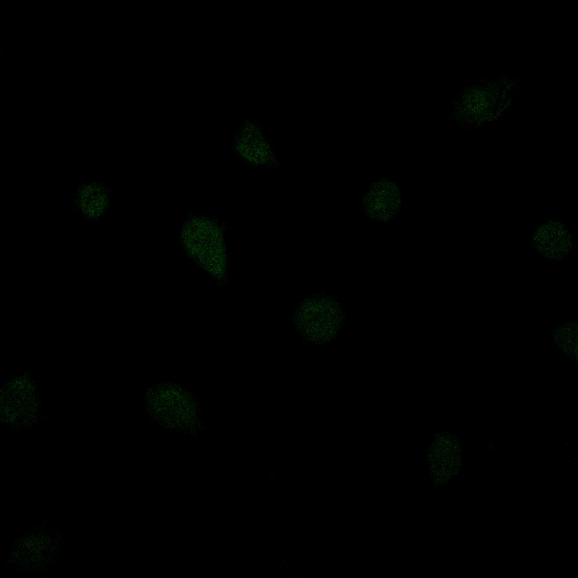

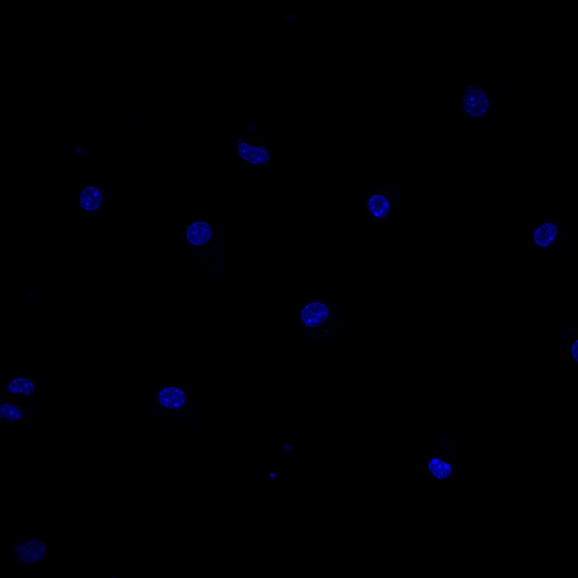

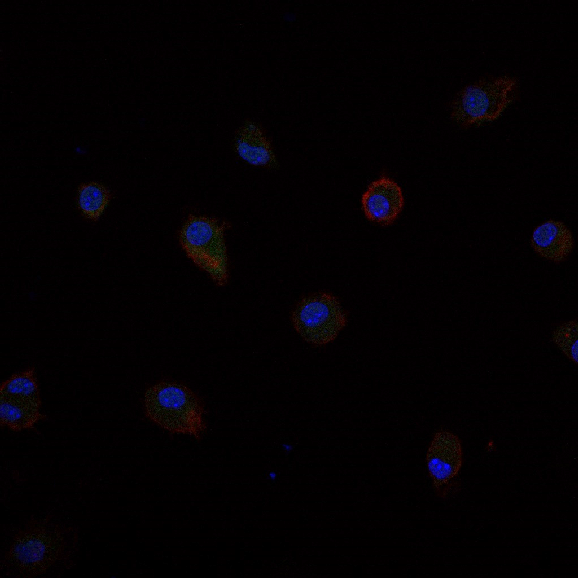

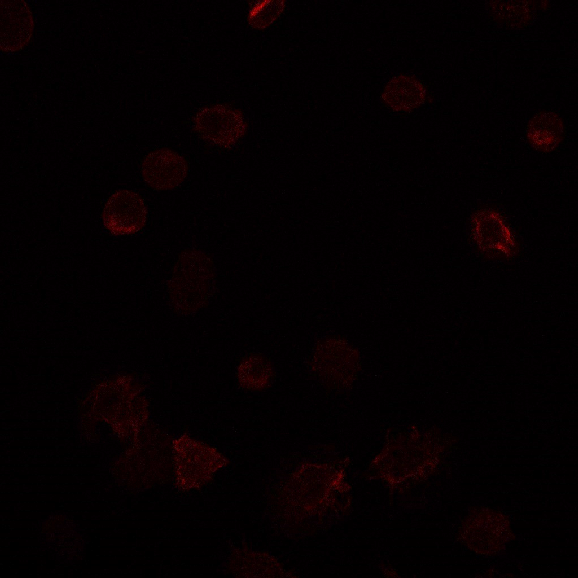

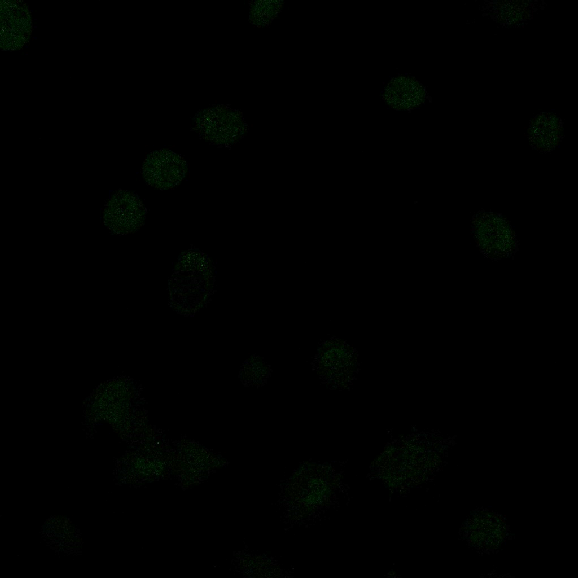

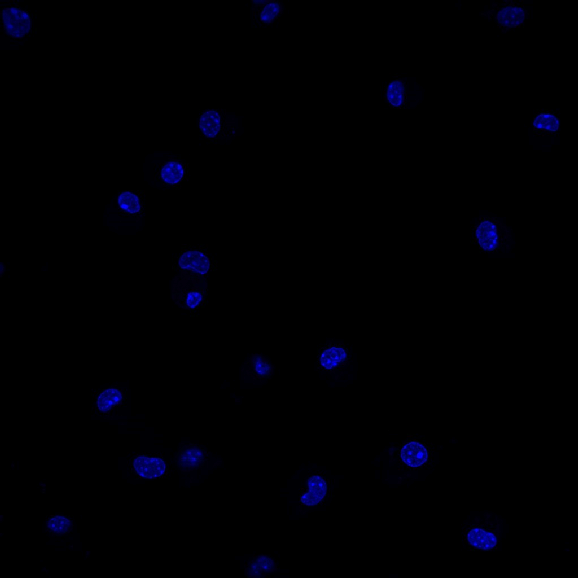

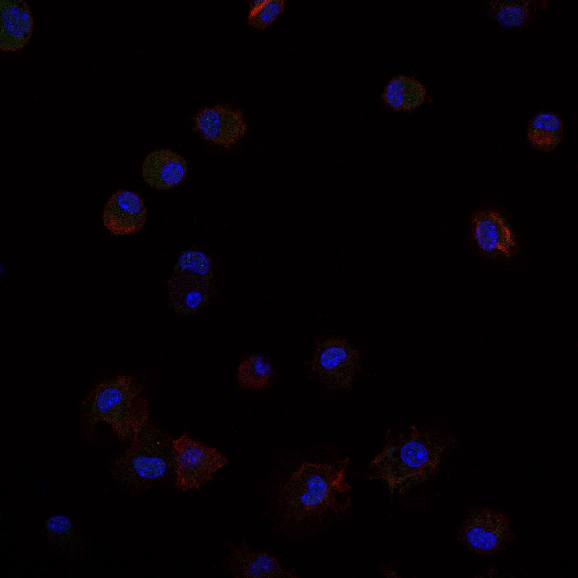


D-Mannitol

control siRNA

control

HG

TAB1 siRNA

HG+TAB1 siRNA

F4/80 P65 DAPI Merge


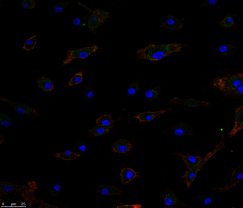

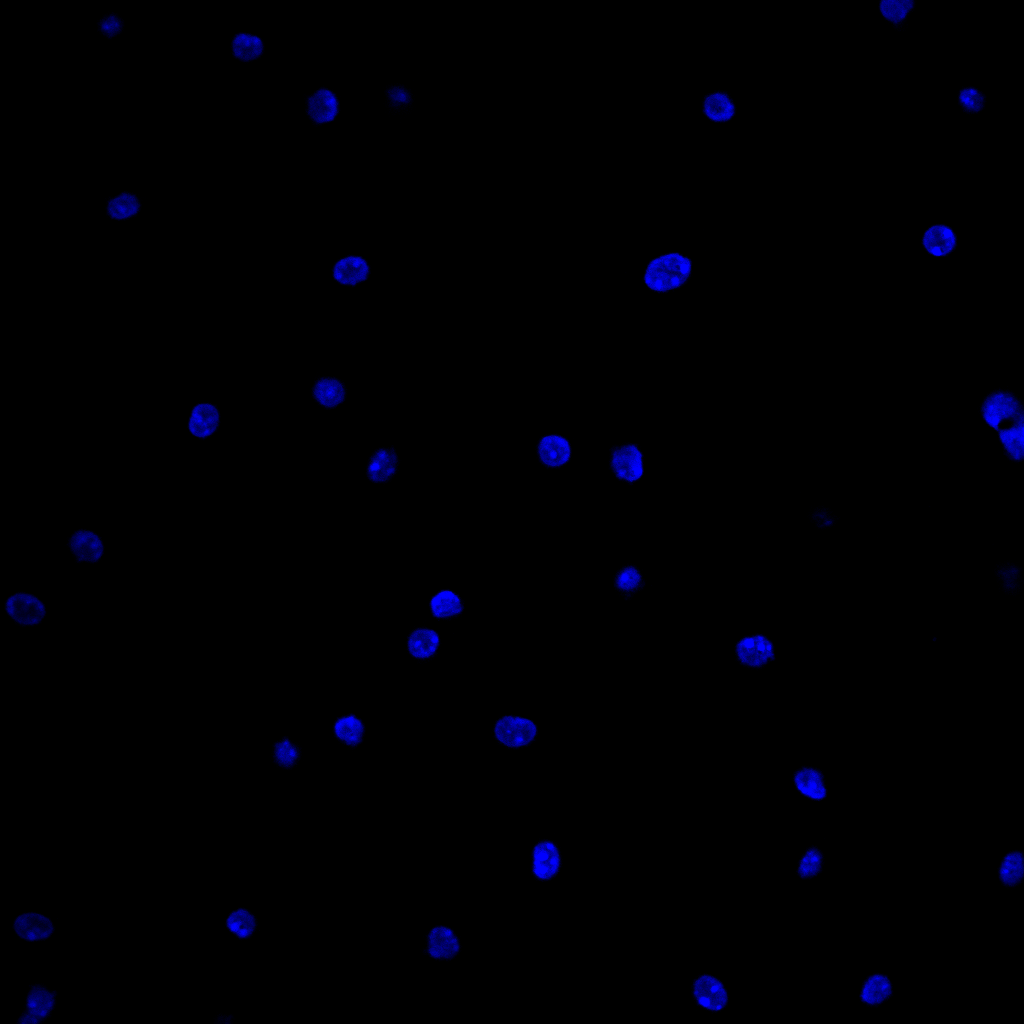

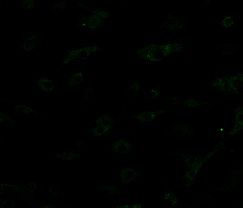

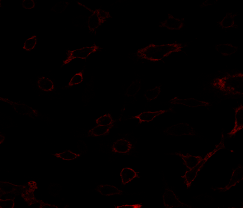


D-Mannitol

control siRNA

control

HG

TAB1 siRNA

HG+TAB1 siRNA

F4/80 iNOS DAPI Merge
